# The responses of cancer cells to PLK1 inhibitors reveal a novel protective role for p53 in maintaining centrosome separation

**DOI:** 10.1038/s41598-017-16394-2

**Published:** 2017-11-23

**Authors:** Linda Smith, Raed Farzan, Simak Ali, Laki Buluwela, Adrian T. Saurin, David W. Meek

**Affiliations:** 1 Division of Cancer Research, Medical Research Institute, Ninewells Hospital and Medical School, The University of Dundee, Dundee, DD1 9 SY United Kingdom; 2 Department of Surgery and Cancer, Imperial College London, Hammersmith Hospital Campus, London, W12 0NN United Kingdom

## Abstract

Polo-like kinase-1 (PLK1) plays a major role in driving mitotic events, including centrosome disjunction and separation, and is frequently over-expressed in human cancers. PLK1 inhibition is a promising therapeutic strategy and works by arresting cells in mitosis due to monopolar spindles. The p53 tumour suppressor protein is a short-lived transcription factor that can inhibit the growth, or stimulate the death, of developing cancer cells. Curiously, although p53 normally acts in an anti-cancer capacity, it can offer significant protection against inhibitors of PLK1, but the events underpinning this effect are not known. Here, we show that functional p53 reduces the sensitivity to PLK1 inhibitors by permitting centrosome separation to occur, allowing cells to traverse mitosis and re-enter cycle with a normal complement of 2N chromosomes. Protection entails the activation of p53 through the DNA damage-response enzymes, ATM and ATR, and requires the phosphorylation of p53 at the key regulatory site, Ser15. These data highlight a previously unrecognised link between p53, PLK1 and centrosome separation that has therapeutic implications for the use of PLK1 inhibitors in the clinic.

## Introduction

p53 is a short-lived transcription factor that is activated and stabilized in response to a range of cancer-relevant stress stimuli including DNA damage, hyper-proliferation, and hypoxia^1–3^. Activated/induced p53 orchestrates changes in gene expression leading to tumour suppressive outcomes of growth arrest (transient or permanent) or programmed cell death. Importantly, p53 also has homeostatic functions, such as control of stem cell renewal and regulation of intermediary metabolism, which may also contribute to tumour suppression^3,4^.

Cells experiencing impairment of the mitotic machinery can undergo apoptosis within mitosis (resulting from mitotic “catastrophe”), while others eventually escape the spindle assembly checkpoint, abort mitosis, and re-enter G1 with abnormal ploidy^5^. No direct role for p53 has been identified within mitosis itself. However, it is clear that p53 can respond to disruption to mitotic integrity following mitotic exit, at which point it can stimulate cell death or senescence as a means of preventing the survival of cells with chromosomal instability^5,6^. Cells failing to undergo normal mitotic progression accumulate *de novo* DNA damage, leading to activation of the protein kinases ATM (ataxia-telangiectasia mutated) and ATR (ATM- and Rad3-related) and, consequently, post-mitotic phosphorylation and activation of p53^6–11^. Cells encountering centrosomal impairment can also undergo delays in mitosis, with similar abortive outcomes^12^. Additionally, p53 controls the levels of Aurora A, an upstream component of the protein kinase cascades responsible for the timely disjunction and bidirectional movement of the centrosomes^13,14^.

PLK1 is a member of the polo-like kinase (PLK) family that mediates several key functions throughout mitosis including centrosome disjunction and movement, activation of cyclin B/CDK1, spindle assembly, and cytokinesis^15,16^. Consistent with these roles, inhibition of PLK1 arrests cells in early mitosis with a characteristic polo “ring” of chromosomes undergoing monopolar attachment to duplicated but unseparated centrosomes. More recently, PLK1 has also been linked to roles in DNA replication^17,18^. PLK1 levels are tightly regulated over the course of the cell cycle^19–21^ and its protein kinase activity is activated through phosphorylation by Aurora A^22,23^. *PLK1* expression is down-regulated by p53 as part of the G2/M checkpoint^24–26^ and its levels are elevated in a range of different tumour types, especially where p53 function has been lost^27^.

PLK1 is considered to be a highly promising cancer therapeutic target and several PLK1 inhibitors have shown promising results in clinical trials to date^20,28–30^. Several laboratories have reported that cancer cells lacking wild type p53 are significantly more sensitive to PLK1 inhibition as compared with cells retaining wild type p53 function^26,31–35^, suggesting that p53 can offer protection against PLK1 inhibitors. Importantly, this outcome has been established in a variety of cellular backgrounds^32,35^, and raises the possibility, from a therapeutic perspective, that cancers retaining wild type p53 may be less responsive to agents targeting PLK1. However, the mechanism(s) underpinning this apparent protective role of p53 remains unclear.

In the present study we show that, following treatment with either of two independent PLK1 inhibitors, GSK461364^36^ and BI6727 (volasertib)^37^, p53-competent cells, but not p53-null cells, can survive and re-enter cell cycle with a normal complement of 2N chromosomes. Underpinning this effect, we find that the early mitotic delay induced by PLK1 inhibitors is significantly less in cells expressing wild type p53 which, unlike p53-null cells, are able to maintain the integrity of centrosome movement. These results highlight a novel p53-mediated compensatory pathway that can maintain cell integrity by overcoming impairment of mechanisms underpinning early mitosis, but which may work adversely from a therapeutic perspective.

## Results

### Wild type p53 protects cells from death induced by PLK1-targeted inhibitors

**Figure 1:**
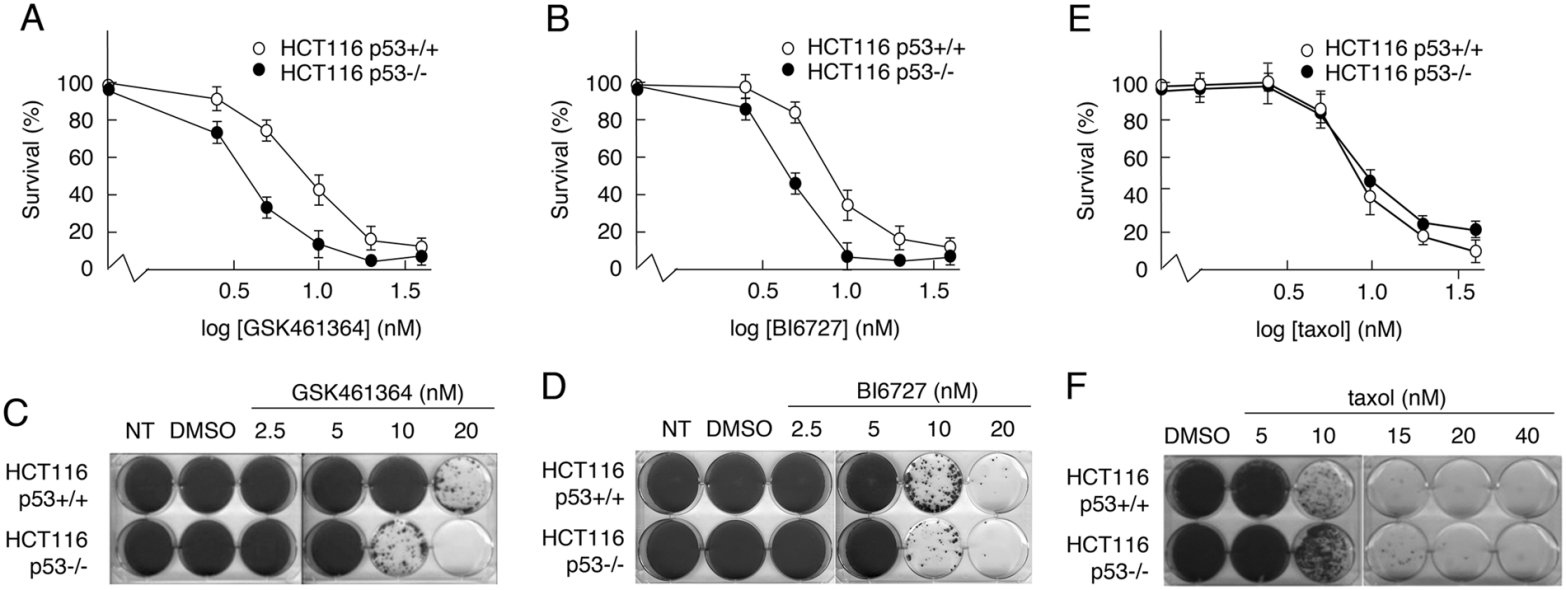
Cells expressing wild type p53 show reduced sensitivity to inhibitors, GSK461364 and BI6727. (**A**,**B**,**E**) Parental HCT116 (p53+/+) cells and a derivative line lacking p53 expression (p53−/−) were treated for 72 h with increasing concentrations of the PLK1 inhibitors, GSK461364 (**A**) or BI6727 (**B**), or with taxol (**E**). Cell viability was then measured using an MTS assay. (**C**,**D**,**F**) HCT116-p53+/+ or -p53−/− cells treated for 72 h with increasing concentrations of GSK461364 (**C**), BI6727 (**D**) or taxol (**F**). Following removal of drugs, cells were grown for a further 12 days. Surviving colonies were detected by staining with crystal violet. Data are representative of three independent experiments each conducted in duplicate.

Several reports have suggested that PLK1-targeted drugs may be less effective towards cancer cells that retain p53 function^26,31–35^. To confirm these observations, the effects of two independently developed, commercially available PLK1 inhibitors, GSK461364 and BI6727, were measured in cell viability (MTS) assays using HCT116 cells (which express wild type p53) and a derivative line with a targeted deletion of the *TP53* gene^38^. The data (Fig. 1A,B) confirmed that, while both GSK461364 and BI6727 reduced the viability of cells in a dose-dependent manner, in each case cells expressing wild type p53 were significantly less sensitive to the drug treatment. These data are consistent with previous observations^26,31–35^. Given that the drugs were developed independently by different companies, it is unlikely that the outcomes of treatment resulted from off-target effects.

Responses were also measured using a colony-survival assay, which was conducted in a dose-dependent manner to provide a comparative indication of the minimum concentration required in each case to give rise to colonies and an assessment of the decrease in the number of colonies as the drug level increases incrementally. The data from these analyses confirmed that the cells were sensitive to GSK461364 and BI6727 in a dose-dependent manner (Fig. 1, panels C and D respectively). As with the MTS assay, cells expressing wild type p53 appeared to be less sensitive to drug-induced loss of survival, again highlighting a possible protective function for wild type p53.

To determine whether the differential sensitivity of the p53+/+ and p53−/− cells is a general feature of mitotic inhibitors, or whether it is specific to PLK1 inhibitors, cells were treated with increasing concentrations of taxol. Both MTS assays (Fig. 1E) and colony formation assays (Fig. 1F) indicated that p53 did not contribute significantly to HCT116 cell viability following treatment with taxol.

### Cells with a normal complement of 2N DNA are detectable following treatment with PLK1 inhibitors, but only when wild type p53 is present

**Figure 2:**
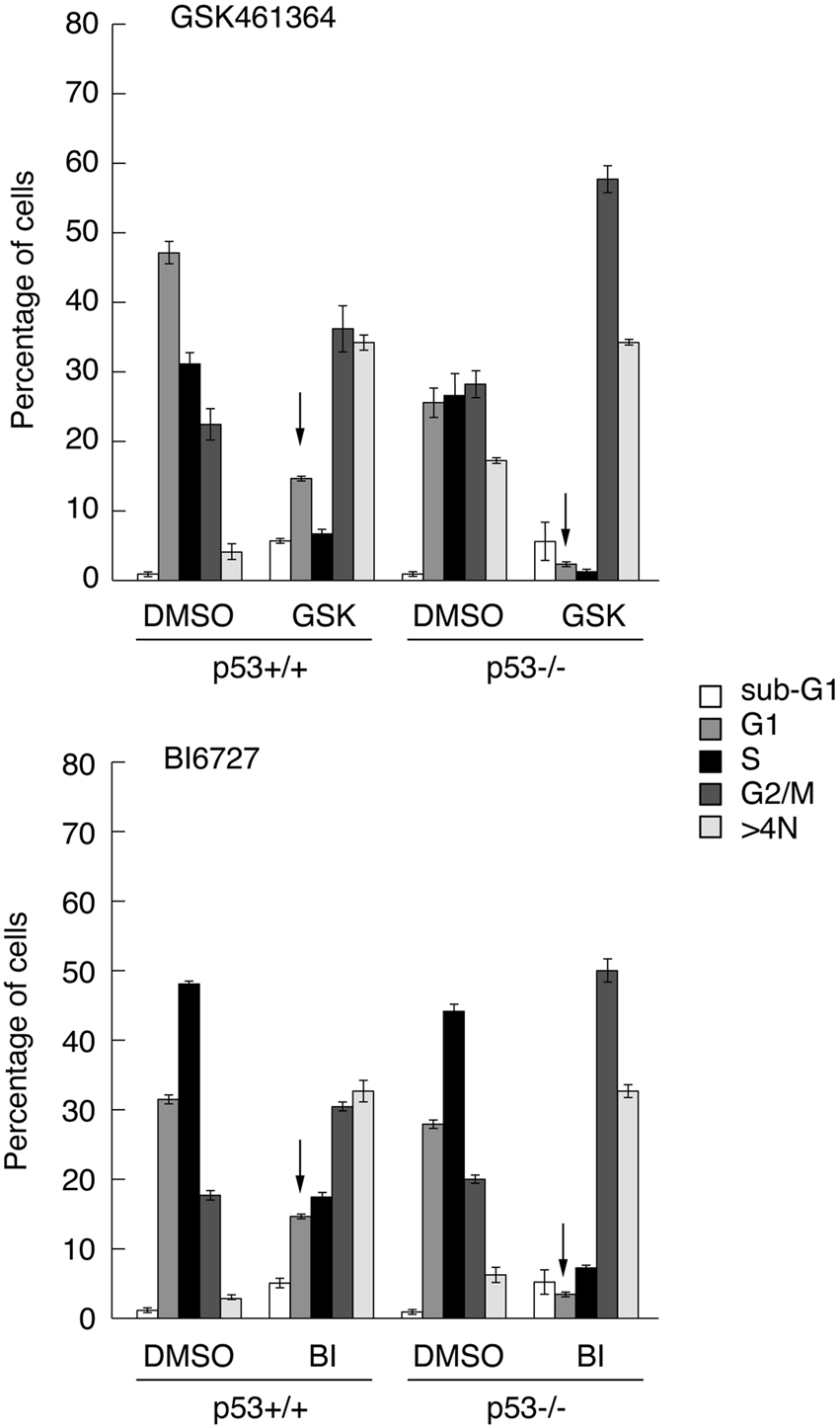
Cells expressing wild type p53 show a partial G1 arrest in response to GSK461364 or BI6727. HCT116-p53+/+ or -p53−/− cells were treated for 24 h with GSK461364 (20 nM) or BI6727 (10 nM) or with DMSO as control. Cells were subsequently harvested and analyzed by flow cytometry. Arrows highlight increased number of p53+/+ cells in G1 following drug treatment as compared with the p53−/− cells. The data are representative of two independent experiments, each done in triplicate. Error bars represent the standard deviation of the mean.

To determine how p53 can mediate a differential response to PLK1 inhibitors, HCT116-p53+/+ and -p53−/− cells were treated with 20 nM GSK461364 or 10 nM BI6727 for 24 h and analyzed by flow cytometry (Fig. 2). Untreated cells showed a standard cell cycle distribution that was unaffected following treatment with DMSO alone. Upon treatment with GSK461364 the largest proportion of p53−/− cells was in G2/M (4N) with significant fractions at 8N (suggesting endo-reduplication) and sub-G1 (<2N, suggesting apoptosis) (Fig. 2). While these same fractions were also observed in the parental (p53+/+) cells, there was an additional population of cells at 2N representative of cells in G0/G1. Similar data were obtained when the cells had been treated with BI6727 in place of GSK461364 (Fig. 2).

The analysis was also conducted in MCF7 and U2OS cells following silencing or mock-silencing of p53. The data confirmed that the presence of cells in G1 was dependent on p53 and that it was not a cell line-specific response (Supplementary Fig. 1). (It should also be note that endo-reduplication was observed only in the HCT116 cells).

### The presence of 2N G1 cells after treatment occurs post-mitotically

**Figure 3:**
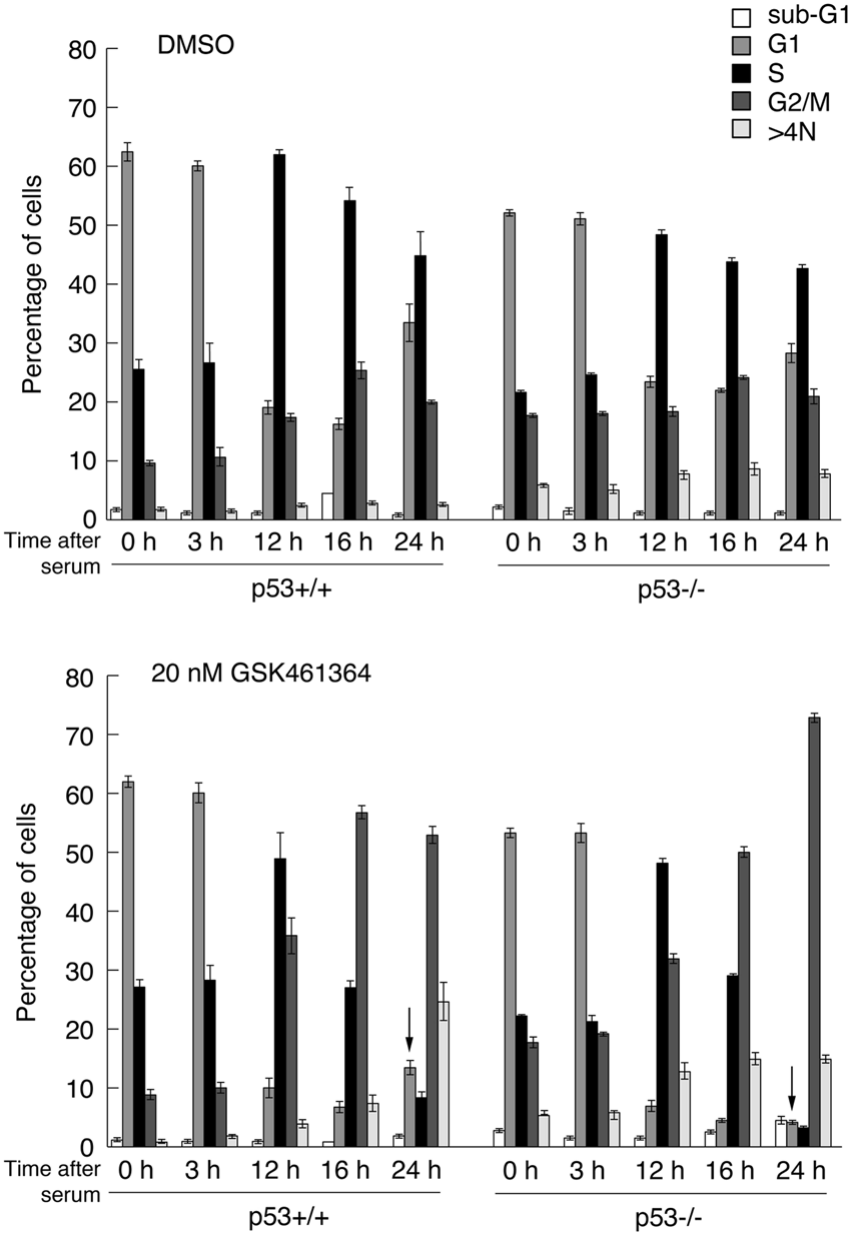
Treatment of synchronized HCT116 cells with GSK461364 permits re-entry into G1 with 2N DNA only if p53 is present. HCT116-p53+/+ or -p53−/− cells were arrested in 0.1% serum then stimulated to re-enter cell cycle by addition of 10% FBS. Treatments with DMSO (as control, upper panel) or GSK461364 (20 nM, lower panel) were initiated 1 h after serum stimulation. Cells were harvested for flow cytometry analysis at indicated times. Arrows (bottom panel) highlight the re-appearance of cells in G1 in the p53+/+ cells, but not in the p53−/− cells. The data are representative of two independent experiments, each done in triplicate. Error bars represent the standard deviation of the mean.

The presence of cells with 2N DNA following treatment with the PLK1 inhibitors could result from the arrest of cells in G1 phase at the time of treatment or, alternatively, from cells entering mitosis and undergoing cytokinesis. To distinguish between these two possibilities, HCT116 (p53+/+ and p53−/−) cells were arrested in G0 by serum withdrawal for 24 h, then stimulated to re-enter cycle synchronously by the re-addition of serum. 1 h after serum addition the cells were treated with 20 nM GSK461364 and analyzed by flow cytometry over a 24 h period. The profiles of p53+/+ and p53−/− cells were essentially indistinguishable up until 16 h post-treatment at which point the cells were almost exclusively 4N (Fig. 3). At 24 h post-treatment, however, a significant proportion of p53+/+, but not p53−/−, cells had re-entered cycle with the normal complement of 2N DNA. These data suggest that p53 permits at least a proportion of treated cells to transit mitosis, undergo cytokinesis, and re-enter G1 phase.

### PLK1 inhibitors induce phosphorylation and activation of p53 via enzymes in the DNA damage pathways

Western blotting analysis of GSK461364- or BI6727-treated parental HCT116 cells and p53−/− derivatives indicated that the levels of PLK1 were significantly elevated at 24 h post-treatment in both lines (Supplementary Fig. 2A–C), consistent with arrest at G2/M, at which stage PLK1 levels are naturally elevated^21^. The coincident appearance of phosphorylated histone H3 confirmed arrest in M phase and not in G2. At 48 and 72 h PLK1 levels had declined suggesting that the cells had largely aborted mitotic arrest. The changes in PLK1 protein levels were independent of the presence of p53. Similar observations were made using MCF7 and U2OS cells (Supplementary Fig. 2D and E respectively) indicating that these effects were not cell line-dependent. Silencing of p53 in the U2OS cells confirmed that changes in PLK1 levels were independent of the presence of p53 (Supplementary Fig. 2E). Treatment of the parental HCT116, MCF7 or U2OS cells with either of the PLK1 inhibitors led to the induction of p53, p21 (a marker for p53 activity), and phosphorylated H2AX (γ-H2AX) (all evident by 24 h: Supplementary Fig. 2), suggesting that p53 may be induced through the DNA damage response. The late induction of these proteins fits with the well-established model that p53 responds to impairment of mitotic events after mitosis has been aborted^6^.

**Figure 4:**
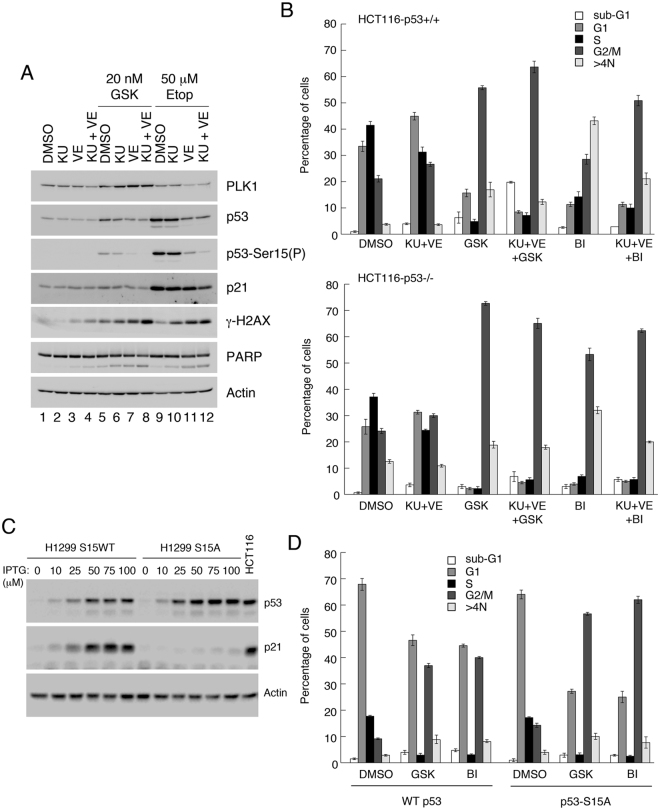
Induction of p53 and appearance of post-mitotic G1 (2N) cells by PLK1 inhibitors occurs through the DNA damage response pathways. (**A**) HCT116-p53+/+ cells were pre-treated for 1 h with 10 μM KU55933 and/or 10 μM VE821, and subsequently treated for 24 h with 20 nM GSK461364 or, as control, 50 μM etoposide. Cell extracts were analyzed by western blotting. (**B**) HCT116-p53+/+ and -p53−/− cells were pre-treated for 1 h with 10 μM KU and 10μM VE821 then further treated for 24 h with 20 nM GSK461364 or 10 nM BI6727, or with DMSO as control. Cells were then harvested and analyzed by flow cytometry. (**C**) H1299 (endogenous p53-null) cells ectopically expressing wild type p53, or a S15A substitution mutant of p53, via the LacSwitch II system (Stratagene) were treated for 16 h with increasing levels of the inducer, IPTG. Cell extracts were analyzed by western blotting as indicated. (**D**) H1299-WTp53 or H1299-S15A-p53 cells were treated for 16 h with 100 μM IPTG, followed by treatment for 24 h with 20 nM GSK461364 or 10 nM BI6727, or DMSO as control. Cells were harvested and analyzed by flow cytometry. Panels A and C show cropped western blots: full length gels including molecular weight markers are provided in the Supplementary Information. The data in panels B and D are each representative of two independent experiments, each done in triplicate. Error bars represent the standard deviation of the mean.

To explore whether protection against PLK1 inhibitors also required activation of p53 through DNA damage signaling, HCT116-p53+/+ cells were treated with GSK461364 for 24 h. Western blotting showed that phosphorylation of Ser15, a marker for p53 induction by DNA damage, increased over-and-above the relative increase in p53 protein levels (Fig. 4A: compare lanes 1 [mock-treated] and 5 [GSK461364-treated]). As a positive control the cellular response to etoposide was included for comparison (lane 9). To determine whether GSK461364-induced Ser15 phosphorylation was dependent on ATM and ATR, which phosphorylate p53-Ser15 in response to DNA strand breaks^39^, cells were pretreated either singly or in combination with inhibitors of the two kinases, KU55933 and VE821 respectively, prior to PLK1 inhibition (lanes 6–8). The data indicate that, while inhibition of either ATM or ATR impaired Ser15 phosphorylation, the combined use of both inhibitors eliminated this post-translational modification, decreased p53 induction and decreased p21 expression. (A similar effect was seen following etoposide treatment, as a positive control). These data support the idea that PLK1 inhibition activates p53 through the DNA damage pathways.

Further analysis by flow cytometry revealed that the fraction of 2N G1 cells that arose following PLK1 inhibition only in p53-competent cells, could also be eliminated through pretreatment with the ATM and ATR inhibitors (Fig. 4B). These observations support the idea that p53 induced by PLK1-mediated activation of DNA damage pathways permits cells to traverse cytokinesis and re-enter cycle with 2N DNA.

To confirm the role of DNA damage signaling pathways in activating p53 following PLK1 inhibition, the responses of clones of H1299 (p53-null) cells inducibly-expressing homeostatic levels of wild type p53 or a p53-S15A substitution mutant were examined^40,41^. Loss of the Ser15 phosphorylation site, which is required to induce p53-target genes^40^ (Fig. 4C), led to a significant reduction in the appearance of cells with 2N DNA following impairment of PLK1 activity (Fig. 4D).

Activation of p53 following treatment with PLK1 inhibitors could occur as: (i) a direct result of DNA damage caused by inhibition of PLK1^18,42^; (ii) an off-target effect of the drugs; or (iii) could simply reflect cell death induced by these compounds. Silencing *PLK1* expression confirmed that loss of PLK1 gave rise to a γ-H2AX signal (Supplementary Fig. 3), indicating that the DNA damage was unlikely to be an off-target effect. Moreover, pre-treatment with the pan-caspase inhibitor, Z-VAD-FMK, for 1 h prior to addition of PLK1 inhibitors, effectively blocked apoptosis but did not impair the DNA damage signal induced by the PLK1 inhibitors (Supplementary Fig. 4A,B). Taken together, these data strongly suggest that the occurrence of DNA damage is a direct effect of PLK1 inhibition and is responsible for activating p53.

### p53 reduces mitotic delay following treatment with PLK1 inhibitors, but not taxol

**Figure 5:**
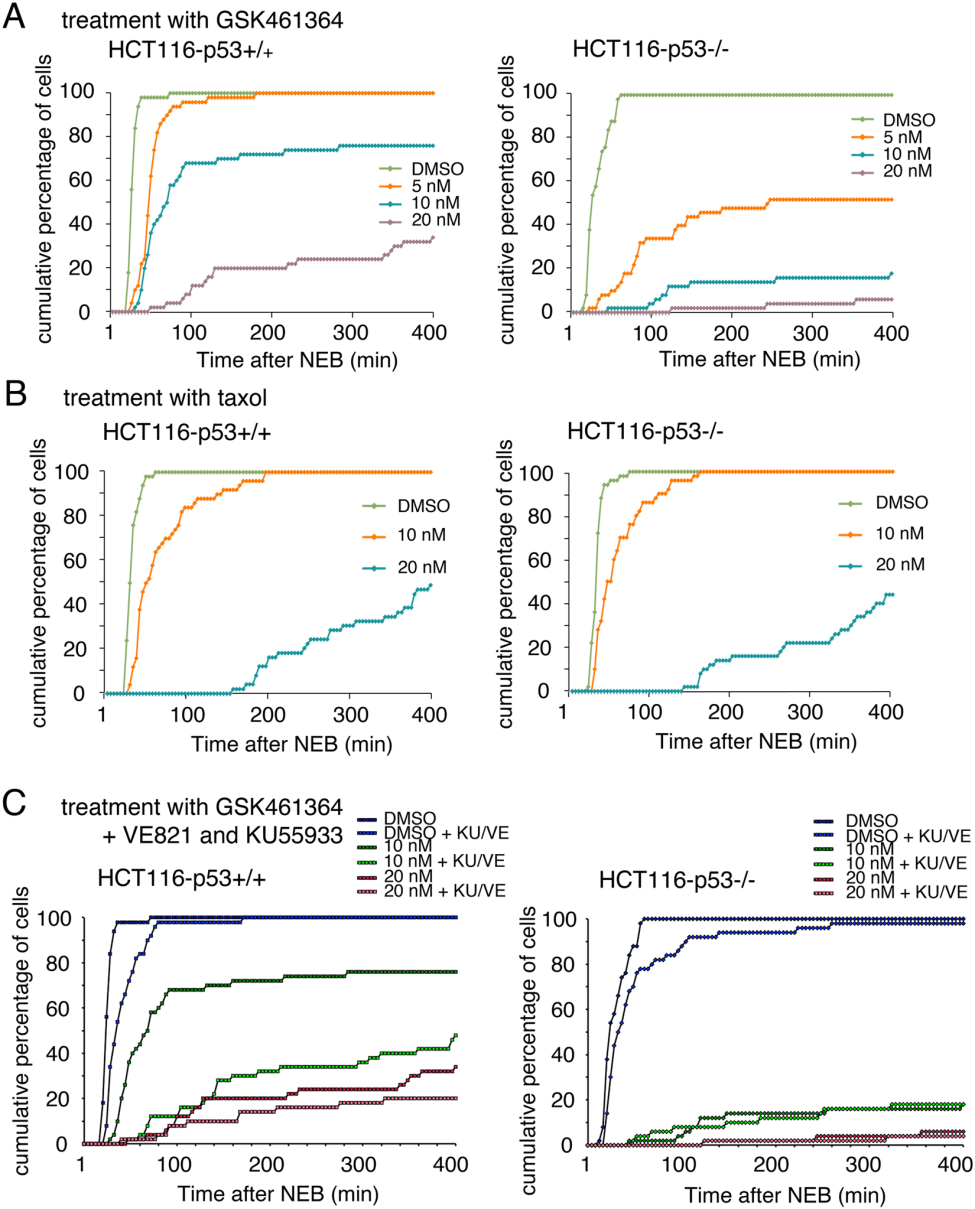
p53 reduces the delay in mitosis resulting from inhibition of PLK1. HCT116-p53+/+ (left panels) and -p53−/− cells (right panels) were treated with DMSO, 5, 10 or 20 nM GSK461364 (**A**) or DMSO, 10 or 20 nM Taxol (**B**). Time-lapse microscopy analysis was then used to determine the duration of mitosis. (**C**) HCT116-p53+/+ and -p53−/− cells were pretreated for 1 h with DMSO or 10 μM KU-55933 and 10 μM VE-82, then subsequently treated with DMSO, 10 or 20 nM GSK461364. Time-lapse analysis was used to determine the duration of mitosis. Graphs show cumulative data from 50 cells for each treatment and are representative of three replicates.

To explore further how p53 might permit PLK1-inhibited cells to re-enter cycle with 2N DNA the abilities of p53-competent and -null cells to traverse mitosis following PLK1 inhibition was compared by time-lapse microscopy. As a control, the responses to mitotic arrest with taxol were assessed. Treatment with increasing concentrations of GSK461364 led to progressively longer delays of the p53-competent cells in traversing mitosis (Fig. 5A). While qualitatively similar, the drug was significantly more potent in delaying mitotic progression of p53-null cells. These data suggest that p53 can mitigate mitotic arrest arising from PLK1 inhibition. When cells were treated with taxol in place of GSK461364, the dose-dependent delay of p53+/+ and p53−/− cells in traversing mitosis was essentially indistinguishable (Fig. 5B), indicating that the p53 response is selective to PLK1 inhibition. When MCF7 cells, and a derivative line in which p53 had been deleted using CRISPR, were treated with the PLK1 inhibitors and analyzed in a manner similar to HCT116 cells, cells lacking p53 were again delayed significantly longer in mitosis than parental cells, confirming that the response was not cell line-dependent (Supplementary Fig. 5).

To determine whether the mitotic delay caused by PLK1 inhibition was associated with DNA damage pathways, the time-lapse analysis with the HCT116 cells was repeated in the presence of the ATM and ATR inhibitors (KU55933 and VE821 respectively). These drugs significantly extended mitotic delay in p53-competent cells but not in p53-null cells (Fig. 5C), suggesting that the ability to limit PLK1 inhibitor-dependent mitotic delay is dependent upon activation of p53 by DNA damage pathways.

### p53 protects cells from PLK1 inhibition by maintaining centrosome separation

**Figure 6:**
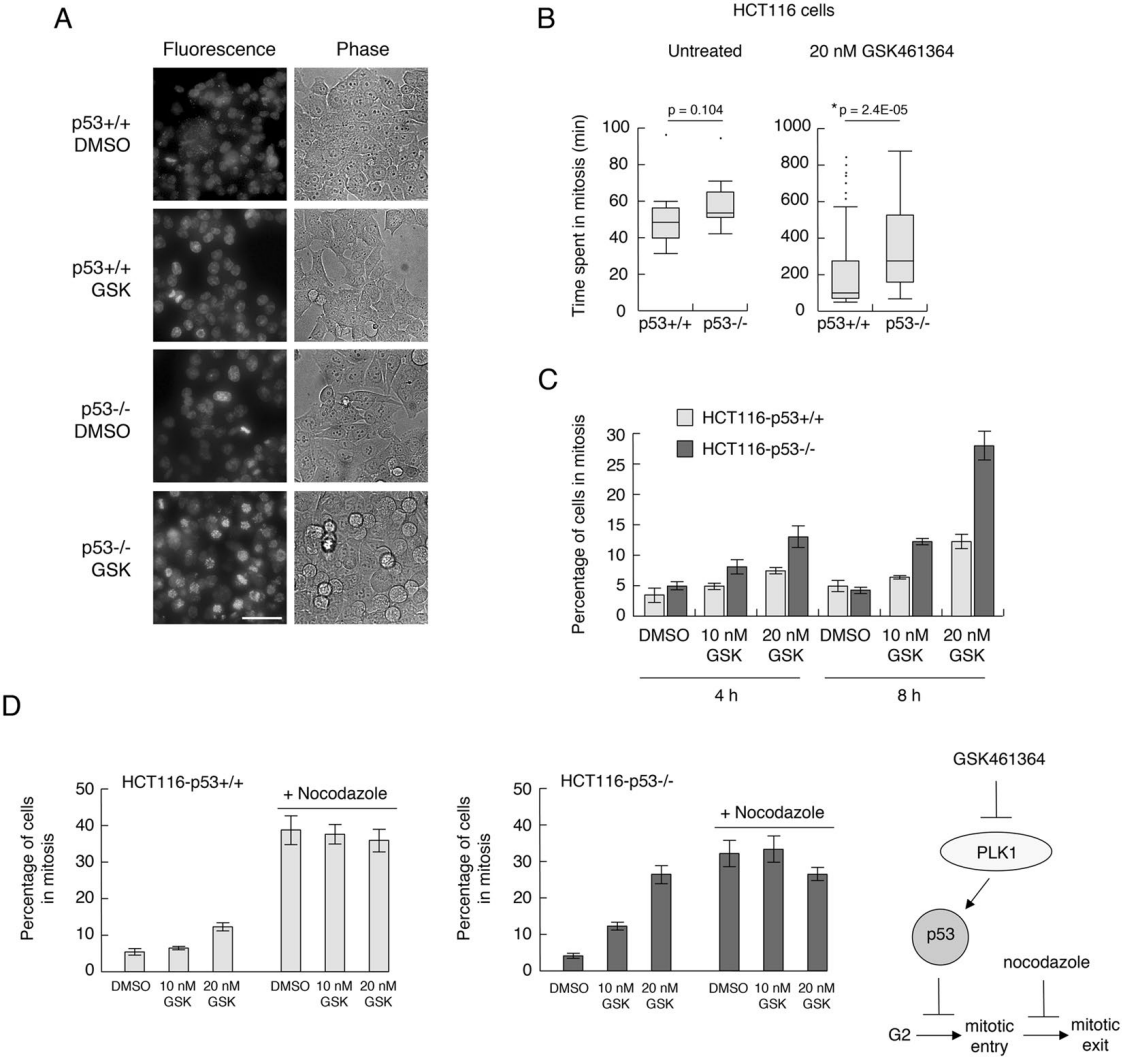
Cells lacking p53 are delayed in mitosis following treatment with GSK461364 (**A** and **B**) HCT116-p53+/+ and -p53−/− cells were stained with SiR-DNA (SiR-Hoechst*) and treated with DMSO or 20 nM GSK461364. Cells were mounted on the Deltavision Elite live cell microscope and imaged using the Cy5 (far red) channel and in phase contrast every 4 minutes over 250 time points. (**A**) representative images of the final time points (16.6 h post-treatment). The scale bar (shown in the bottom left hand image) represents 50 microns. (**B**) left hand plot: the lengths of time spent in mitosis for 22 HCT116-p53+/+ and 22 HCT116-p53−/− cells are presented as box plots (showing median and quartiles). The data from 77 HCT116-p53+/+ and 77 HCT116-p53−/− GSK461364-treated cells is shown in the right hand plot. (**C**) HCT116-p53+/+ and -p53−/− cells were treated with DMSO, 10 nM or 20 nM GSK461364 for 4 and 8 h. Cells were fixed with paraformaldehyde, stained with phosphorylated histone H3 (serine 10) antibody and propidium iodide. Cells were analyzed by flow cytometry, and the percentage of cells positive for phosphorylated histone H3 (serine 10) was plotted as a bar graph. (**D**) HCT116-p53+/+ and -p53−/− cells were treated with DMSO, 10 nM GSK461364, or 20 nM GSK461364, in the absence of presence of 3.3 µM Nocodazole. Cells were subsequently fixed and stained as in panel C. Schematic: by blocking mitotic exit the number of cells in mitosis is a measure of rate of entry into mitosis. The data in panels C and D are representative of two independent experiments. Error bars represent the standard deviation of the mean.

To investigate how p53 can overcome arrest induced by PLK1 inhibitors, further time lapse analysis was conducted using SiR-DNA (SiR-Hoechst*) to visualize the chromosomes (Supplementary Movies 1–4). Representative images from these analyses are shown in Fig. 6A. These data show that all untreated cells examined underwent a normal mitosis and cytokinesis, irrespective of p53 status. Following treatment with 20 nM GSK461364, however, 67% of p53-competent cells entering mitosis could still achieve cytokinesis as compared with only 22% of p53-null cells, most of which failed to achieve metaphase (Supplementary Movies 3 and 4 respectively; Fig. 6A). Moreover, quantitative analysis (Fig. 6B) showed that, while the median amount of time spent by untreated p53+/+ and p53−/− cells in mitosis was indistinguishable, p53−/− cells showed a significantly greater delay in mitosis (median time 280 min) than p53+/+ cells (median time 100 min) after GSK461364 treatment. Phospho-histone staining (Fig. 6C) confirmed that an increasingly larger proportion of p53−/− cells were in mitosis as compared with p53+/+ parental cells, and that this was not the consequence of a differential rate at which cells enter mitosis (Fig. 6D), thereby ruling out a role for p53 in restricting mitotic entry. Taken together, these data suggest that p53 may respond to, and/or protect against, failure of a PLK1-mediated event(s) in early mitosis prior to the formation of a mitotic spindle.

**Figure 7:**
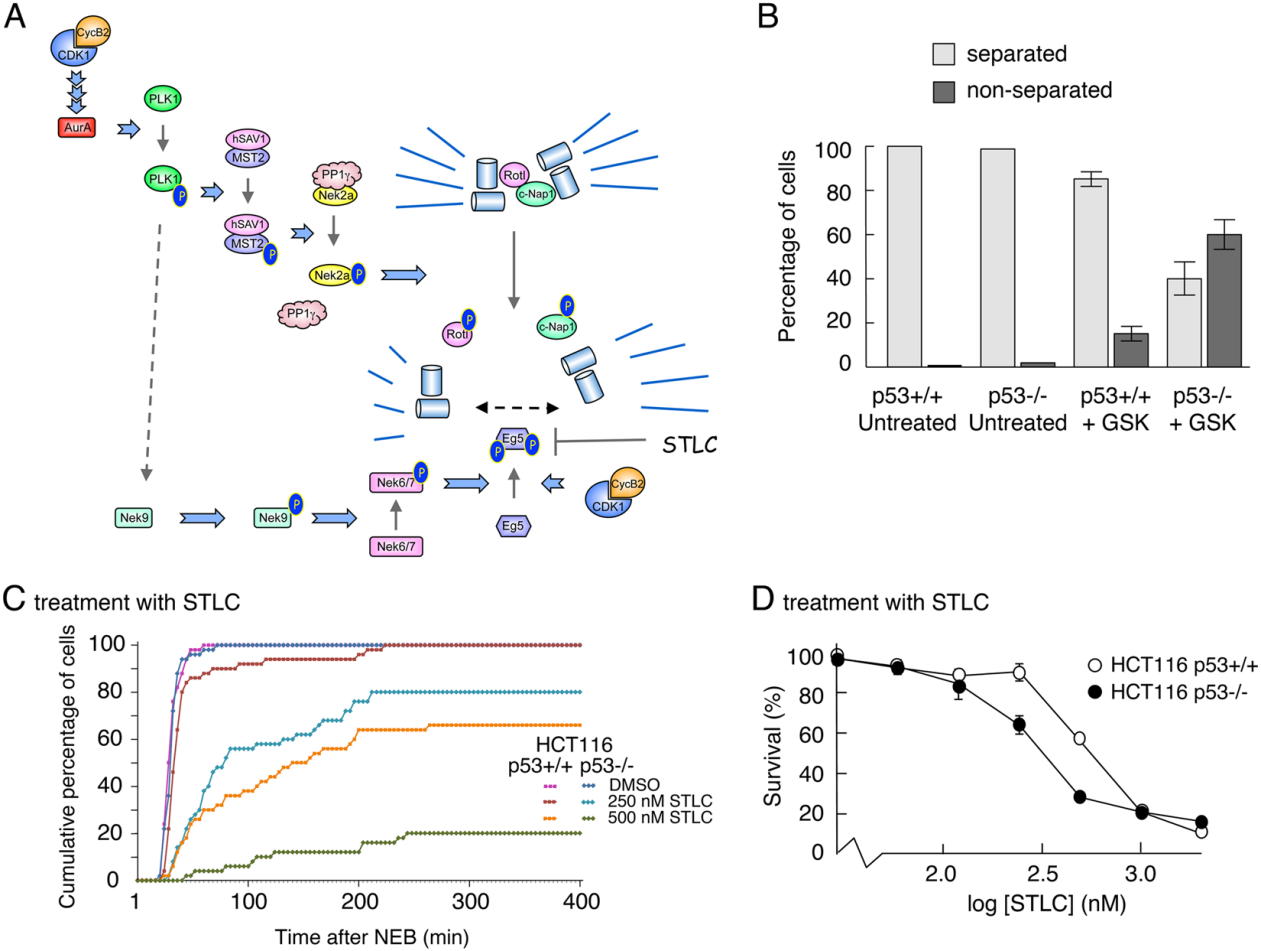
p53 protects cells from inhibition of PLK1 by maintaining centrosome separation. (**A**) Schematic showing the protein kinase cascade responsible for regulating centrosome uncoupling and separation. (**B**) HCT116-p53+/+ and -p53−/− cells were treated with 20 nM GSK461364 or DMSO for 8 h, and subsequently stained for γ-tubulin antibody (a centrosome marker). The number of mitotic cells showing a normal bipolar spindle (separated centrosomes) or monopolar spindle (non-separated centrosomes) was determined using fluorescence microscopy. Over 100 cells were counted for each condition (scored by two independent individuals) and plotted as percentages of separated v non-separated centrosomes (bar graph). (Counting was based on the detection of mitotic cells by use of DAPI, and then switching channels to look at gamma tubulin to assess separation of centrosomes in the mitotic cells). Data are representative of two independent experiments and error bars represent the standard deviation of the mean. (**C**) HCT116-p53+/+ and -p53−/− cells were treated with DMSO, 250 or 500 nM STLC. Time-lapse analysis was then used to determine the duration of mitosis. The graph represents the cumulative data from 50 cells for each treatment and are representative of three replicates. (**D**) HCT116 p53+/+ and p53−/− cells were treated for 72 h with increasing concentrations of STLC. Cell viability was measured using an MTS assay. Data are representative of three independent experiments each conducted in triplicate. Error bars represent the standard deviation of the mean.

PLK1 is a major upstream component of the protein kinase cascades that trigger uncoupling of the duplicated centrosomes and activation of Eg5, the principal kinesin motor that drives centrosomes to opposite poles in the cell^43^ (Fig. 7A). To determine whether p53 has any impact on the impairment of these processes following PLK1 inhibition, HCT116-p53+/+ and -p53−/− cells were treated with GSK461364 followed by fixing and staining with an antibody to gamma-tubulin to visualize the centrosomes. Individual cells were then examined by microscopy to determine whether centrosomal segregation had occurred. Untreated p53-competent and p53-null cells were indistinguishable, showing very little segregation failure. Following GSK461364 treatment, however, only 16% of the p53+/+ cells failed to segregate their centrosomes as compared with greater than 60% of the p53−/− cells at the same level of drug exposure (Fig. 7B and Supplementary Fig. 6). Taken together, these data suggest that p53 may protect cells by ensuring fidelity of centrosome disjunction and/or bidirectional movement.

### p53 safeguards the fidelity of centrosome migration

To determine how p53 might impact on the processes of centrosome separation, HCT116-p53+/+ and -p53−/− cells were treated with increasing concentrations of the Eg5 inhibitor, S-Trityl-L-cysteine (STLC). Measurement of the time spent in mitosis showed that the p53-null cells were significantly more sensitive to the Eg5 inhibitor as compared with the p53-competent cells (Fig. 7C), thereby phenocopying the response to PLK1 inhibitors. Similarly, in an MTS (viability) assay, p53-null cells were significantly more sensitive to STLC than p53-competent cells (Fig. 7D). In contrast, as shown above, the sensitivity of the two lines to taxol was identical in each assay (Figs 1E,F and 5B respectively). These data strongly suggest that p53 can protect against impairment of centrosome separation, but has no effect once spindle formation begins. Moreover, given that separation of the centrosomes must occur *after* their disjunction, it is highly likely that p53 has little impact on the disjunction mechanism itself.

## Discussion

In the present study, we confirm that p53 can offer a selective advantage to cancer cells treated with PLK1 inhibitors. Our analysis indicates that, in response to the PLK1 inhibitors, GSK461364 or BI6727, a proportion of p53-competent, but not p53-null, cells can traverse mitosis and cytokinesis, in a p53- and ATM/ATR-dependent manner, and re-enter cycle with a normal complement of chromosomes.

Our data are consistent with the findings of several laboratories, showing that cancer cells lacking wild type p53 show increased sensitivity to PLK1 inhibition as compared with cells retaining wild type p53 function^26,31–35^. Contrary to these studies, Sanhaji *et al*. have reported that p53 is not directly relevant to the outcome of treating cells with PLK1 inhibitors^44,45^. Although our conclusions regarding biological outcome differ from Sanhaji and colleagues, we do share a number of observations that highlight important differences in the response to PLK1 inhibition arising from the presence or absence of p53. These include: (i) cells lacking p53 show an increased proportion of mono-polar spindles following treatment with BI6727 (and BI2536) as compared with the p53-competent cells^44^, although quantitatively we see a much bigger effect (Fig. 7B, Supplementary Fig. 6); (ii) there is an increased proportion of cells in G1 after PLK1 inhibition when p53 is present^44^ (Figs 2–4); and (iii) there is more DNA damage (as measured by the appearance of γ-H2AX) in the PLK1 inhibitor-treated p53−/− cells as compared with the p53+/+ cells; in our own case we provide evidence this is NOT a direct result of apoptosis [Sup Fig. 4]). Clearly, therefore, while these similarities are evident, it is difficult to be certain regarding the basis on which we reach different conclusions concerning the effects of p53 on cellular fate following treatment with PLK1 inhibitors. One important consideration is perhaps the fact that antimitotic inhibitors can lead stochastically to multiple outcomes within the same population of cells^46^. In the case of PLK1 inhibitors, such a heterogeneous response can be observed from cell cycle profiles (e.g. see Fig. 2) where many cells arrest in M phase, while some undergo apoptosis, some endo-reduplicate (i.e. abort mitosis and re-enter cycle without undergoing cytokinesis leading either to permanent arrest in a G1-like state but with 4N DNA or being able to complete another cycle) and, when p53 is present, some undergo cytokinesis and re-enter cycle with 2N DNA. This heterogeneity is perhaps not surprising given that PLK1 is involved in several key events throughout mitosis. We suggest that such a heterogeneous response may impact upon the interpretation of data. Thus, while largely similar (heterogeneous) outcomes may be observed following drug treatment regardless of the presence of p53, we suggest that protection conferred by p53 may allow a small proportion of cells to survive and it is these that underpin the observed differences in cellular fate between the p53+/+ and p53−/− cells. It is thus entirely plausible that differences between various studies in growth conditions, drug treatment regimes (e.g. concentrations, exposure times etc.) or the types of assay used may either mask or reveal the behaviour of this surviving proportion of cells.

In addition to its well characterized roles in responding to failed mitoses^5,6^, p53 has an earlier role upon entering mitosis in maintaining a balance of the activities in the protein kinase cascade initiated by cyclin B2/CDK1 (Fig. 7A) that is responsible for the timely disjunction and separation of the centrosomes, through regulating the levels of Aurora A^13,14^. We propose here an *additional* involvement for p53 in this pathway in which p53 senses and responds to failure to drive centrosomes to opposite poles of the cell, as indicated by our finding that p53 is required to overcome defects in Eg5 motor activity but has no impact on the subsequent steps of microtubule dynamics. Given that transcription essentially shuts down as cells enter mitosis, this would suggest that either p53 engages in some novel, as yet undefined, transcription-independent function in prometaphase or, more plausibly, that in the presence of p53, a compensatory pathway(s) is already in place before cells enter mitosis, that allows at least a proportion of cells to passage through mitosis and undergo cytokinesis. The observation that p53-competent cells enter and traverse mitosis within a very short time after drug treatment (Supplementary Movie 3), suggests that the p53 “response” to PLK1 inhibitors is relatively fast, and therefore favors the idea that a safety net is already available to ensure proper centrosome dynamics.

The finding that direct inhibition of Eg5 phenocopies the outcomes of treating p53-competent and p53-null cells with PLK1 inhibitors (Fig. 7) suggests that p53 senses impairment of Eg5 activity or some downstream event prior to establishing the spindle. Examination of the levels of Eg5 and Kif15 (motor proteins that are responsible for driving the uncoupled centrosomes to opposite poles of the cell^47–50^) in p53+/+ and p53−/− cells reveals no differences in the homeostatic levels of these proteins (Supplementary Fig. 7), or following stimulation with Nutlin (Supplementary Fig. 8), etoposide (Supplementary Fig. 9), PLK1 inhibitors (Supplementary Fig. 10A–D), or the Eg5 inhibitor, STLC (Supplementary Fig. 10E,F). However, it is possible that p53 may be able to compensate by modulating the levels of Eg5 regulators or partners. While the nature of this compensatory mechanism(s) remains unclear it should be a key focus in developing our understanding of the impact of p53 on maintaining mitotic integrity.

Our data also confirmed previous observations that inhibition of PLK1 can give rise to DNA damage^18,42^ and suggest the possibility that this may be the activating event for p53-mediated protection. Alternatively, however, it is plausible that protection may be provided through basal, unstimulated levels of p53, given that p53-Ser15 phosphorylation, mediated in part by ATM/ATR, can occur homeostatically in the absence of DNA damage^40^.

Finally, our data raise the possibility that cancers retaining wild type p53 may be less responsive clinically to agents targeting PLK1. Given that, from a therapeutic perspective, this could ultimately impact on treatment, it will be important to establish an understanding of the relationship between *TP53* status and the sensitivity of human cancers to PLK1-directed drugs.

## Materials and Methods

### Cell culture

Cells were routinely maintained at 37 °C/5% CO_2_ in Dulbecco’s modified Eagle’s medium (Gibco) supplemented with 10% foetal bovine serum (Biosera) and 2 mM Glutamine (Gibco). MCF7 cells were obtained from ATCC (LGC Standards, UK). HCT116 (p53+/+ and p53−/−) colon carcinoma cells, U2OS osteosarcoma cells, H1299 non-small cell lung carcinoma cells were obtained from the Cancer Research UK Biorepository. Cells were expanded, aliquoted and stored at early passage after purchase/receipt, and were validated for p53 status. Cells were routinely checked for mycoplasma (Mycoalert^TM^, Lonza) and were discarded after passage 16 from thawing.

### Antibodies and reagent

Antibodies were as follows: p53 (DO-1; Moravian Biotechnology), p21 ((H-164): sc-756; SantaCruz Biotechnology), PLK1 (208G4; Cell Signaling Technology), actin (A2066; Sigma), Poly (ADP-Ribose) Polymerase-1 (PARP) (9542; Cell Signaling Technology), γ-H2AX (Phospho-S139) (ab11174; AbCam), p53 (Phospho-S15) (#9284; Cell Signaling Technology), MDM2 (4B2; Moravian Biotechnology), Gamma-tubulin (T65557; Sigma), Histone H3 ((D1H2): 4499; Cell Signaling Technology), Histone H3 (Phospho-S10) (06–570; Millipore), BrdU Pure ((B44): 347580; Becton Dickinson).

PLK1 inhibitors, GSK461364 and BI6727, ATM and ATR inhibitors, KU-55933 and VE-821, and etoposide were from Selleckchem. The specificity and efficacy of GSK461364 and BI6727 have been described previously^36,37^. S-Trityl-L-cysteine (STLC) was from Sigma. Taxol was from LC Labs. Nocodazole was from Millipore.

### Western blot analysis

SDS-PAGE and western blotting was carried out using standard conditions. Images were gathered using a BioRad ChemiDoc^TM^ MP Imaging System using Image Lab Software 4.1. Minor adjustments only were made for background and contrast in Photoshop.

### MTS cell viability assay

2.5 × 10^3^ cells/well were seeded in triplicate in 96-well plates for each condition. The following day cells were left untreated, treated with DMSO (vehicle control) or with drugs as indicated in figure legends. After 68 hours of exposure to the drug, MTS assay (Promega) was performed as directed by the manufacturer.

### Colony formation assay

5 × 10^4^ cells were seeded in 6-well plates. The following day cells were either left untreated (fresh medium added), treated with DMSO (vehicle control), or with drugs as indicated in figure legends.

### Flow cytometry

Cells were fixed in ice-cold ethanol, washed in PBS-1% FBS and treated with 2 M HCl for 20 min at 37 °C, and subsequently with 50 μg/ml Propidium Iodide/200 μg/ml RNase A in PBS. Analysis was performed using a Becton Dickinson FACS Canto Flow Cytometer and FlowJo software.

To discriminate between cells in G2 and M, cells were fixed in 0.5% (w/v) para-formaldehyde for 20 min at 37 °C and incubated with 100 μl of 10 μg/ml anti-phospho-(S10)-histone H3 antibody for 1 h, followed by washing in PBS-1% FBS and incubation with 12.5 μg/ml FITC-labeled goat anti-rabbit IgG for 30 min. Cells were subsequently washed, stained with propidium iodide and analyzed as above.

### Gene silencing

PLK1 and p53 siRNA transfections were performed in 6-well plates using 2.5 × 10^5^ cells and 10 nM siRNA per well, and reverse transfection procedure with Lipofectamine® RNAiMAX (Invitrogen) according to manufacturer’s instructions. Cells were incubated for 24–48 h before harvesting or further treatment.

siRNA oligonucleotides were from Thermo Scientific: p53 (exon 7): 5′-GACUCCAGUGGUAAUCUACUU-3′, (OSLR-001137) PLK1:5′-GCACAUACCGCCUGAGUCU-3′; 5′-CCACCAAGGUUUUCGAUUG-3′; 5′-GCUCUUCAAUGACUCAACA-3′; 5′-UCUCAAGGCCUCCUAAUAG-3′, (L-003290-00) Non-silencing: 5′-CAGUCGCGUUUGCGACUGGUU-3′, (OSLR-001139).

### Live cell imaging

Cells were seeded in 24-well plates to achieve 80% confluency at the time of treatment. After drug treatment imaging was performed in a heated chamber (37 °C, 5% CO_2_) using a 10X/03 objective on a Zeiss Axiovert 200 M microscope controlled by Micro-Manager software. Images were taken every 4 min (20 seconds exposure, 2 × 2 binning) using a C4742-80-12AG camera (Hamamatsu), with 250 images being recorded for each position. Image J software (National Institutes of Health) was used to manually calculate time in mitosis, with start time being the rounding of the cell and end time being the decision of cellular fate (i.e. aborting mitosis or undergoing cytokinesis). For each condition 50 cells were analyzed.

### Live cell imaging (fluorescence)

Cells were seeded in μ-slide 8-well chambers (Ibidi) and left overnight. The following day, the medium was removed and replaced with carbon dioxide-independent Leibovitz’s L-15 medium (Gibco) supplemented with 10% (v/v) FBS and 100 units/ml of penicillin and 100 µg/ml of streptomycin (Gibco). After 4 h, the medium was removed and 100 nM SiR-DNA (tebu-bio), diluted in L-15, was added to the cells for 15 min. Cells were then washed with PBS before addition of L-15 containing the appropriate drugs and mounting on the incubator chamber of a Deltavision Elite microscope fitted with a 40x/1.30NA U Plan FLN oil objective lens, and operated using SoftWoRx software. Images were captured using a CoolSNAP HQ2 camera (Photometrics) (250 time points, 4 min intervals, 4 × 4 binning, 256 × 256 image size, 4 optical sections (5 μm optical spacing).

### Immunofluorescence

Cells were seeded on poly-L-lysine coated coverslips in 24-well plates 24 h prior to drug treatments. Cells were subsequently washed in PBS then fixed with 4% (w/v) para-formaldehyde solution for 15 min. After further PBS washes, cells were permeabilized in 0.1% (v/v) Triton X-100 in PBS for five min. After further PBS washes coverslips were soaked in 5% (w/v) BSA, 0.1% (v/v) Triton X-100 in TBS (blocking solution) for 15 min. Coverslips were then incubated in γ-tubulin antibody diluted in blocking solution for 1 h, followed by washing in PBS, incubation in Alexa Fluor® secondary antibody for 1 h and further washing in PBS. Coverslips were then incubated with DAPI for five min, washed in PBS, and mounted onto slides using ProLong® Gold Antifade Mountant (Life Technologies). Slides were left for 24 h at room temperature and subsequently stored at 4 °C. Samples were examined using a Deltavision Elite microscope fitted with a 40x/1.30NA U Plan FLN or 100X/1.4, UPLS Apo oil objective lens (Olympus), and operated using SoftWoRx software. To measure centrosome separation, mitotic cells were detected detected using DAPI. The γ-tubulin staining was then used to determine visually whether the centrosomes has separated (i.e formed a spindle) or remained in tight proximity at the periphery of the cell. All of these measurements were made independently by two individuals. Representative images were acquired using a Leica DFC420 camera.

## Electronic supplementary material

Supplementary Information

Supplementary Movie 1

Supplementary Movie 2

Supplementary Movie 3

Supplementary Movie 4
