# Author Correction: The responses of cancer cells to PLK1 inhibitors reveal a novel protective role for p53 in maintaining centrosome separation

**DOI:** 10.1038/s41598-018-23384-5

**Published:** 2018-03-22

**Authors:** Linda Smith, Raed Farzan, Simak Ali, Laki Buluwela, Adrian T. Saurin, David W. Meek

**Affiliations:** 1 Division of Cancer Research, Medical Research Institute, Ninewells Hospital and Medical School, The University of Dundee, Dundee, DD1 9 SY United Kingdom; 2 Department of Surgery and Cancer, Imperial College London, Hammersmith Hospital Campus, London, W12 0NN United Kingdom

Correction to: *Scientific Reports* 10.1038/s41598-017-16394-2, published online 23 November 2017

The original version of this Article contained a typographical error in the spelling of the author Adrian T. Saurin, which was incorrectly given as Adrian Saurin. This has now been corrected in the PDF and HTML versions of the Article, and in the accompanying Supplementary Material.

