# Supplementary material for: The responses of cancer cells to PLK1 inhibitors reveal a novel protective role for p53 in maintaining centrosome separation
Source: Sci Rep. 2017 Nov 23;7:16115. doi: 10.1038/s41598-017-16394-2 (PMC5701047; doi:10.1038/s41598-017-16394-2)

SUPPLEMENTARY INFORMATION

The responses of cancer cells to PLK1 inhibitors reveal a novel protective role for p53 in maintaining centrosome separation

Linda Smith, Raed Farzan, Simak Ali, Laki Buluwela, Adrian T. Saurin, and David W. Meek

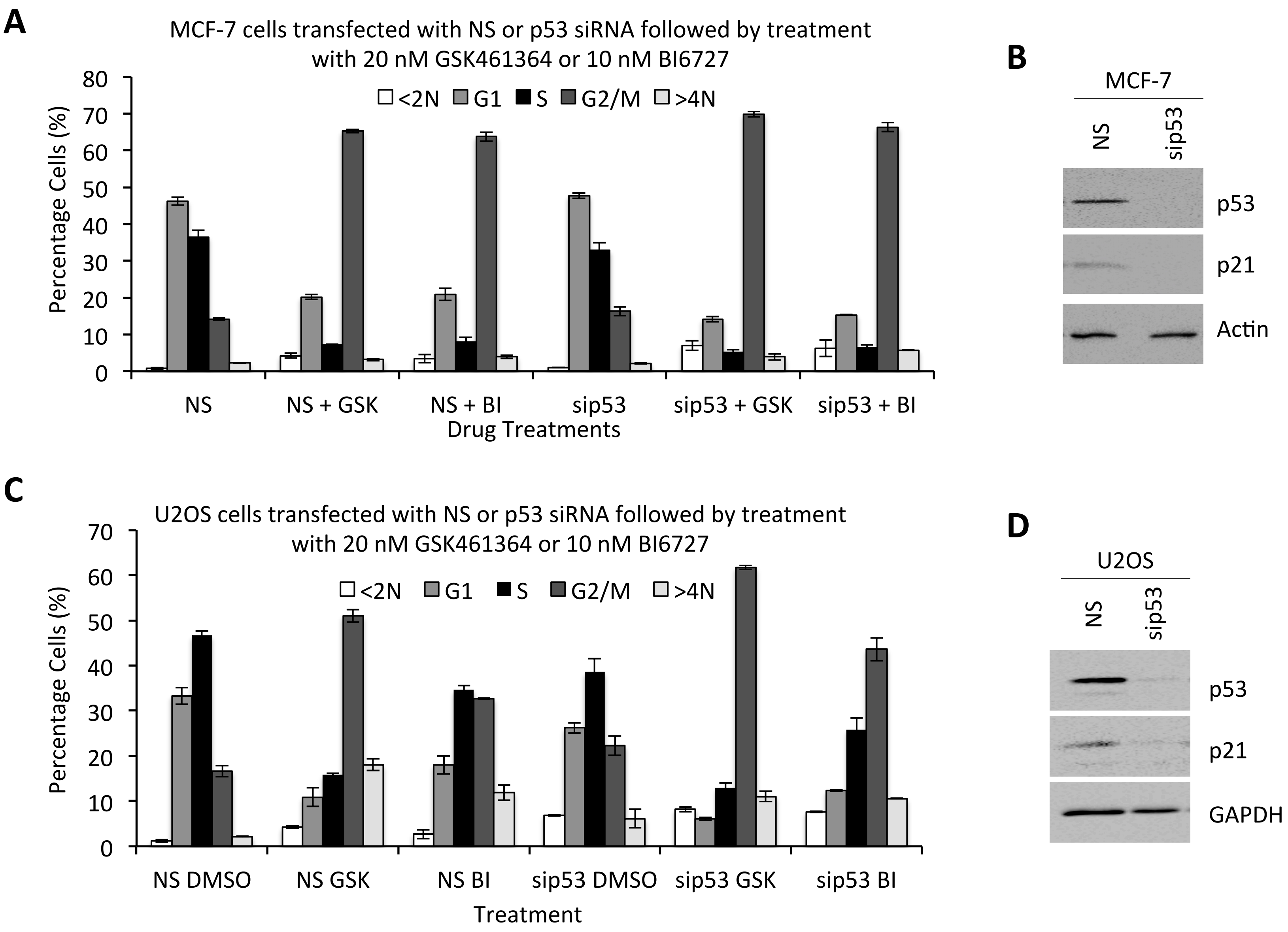

Supplementary Figure 1. Partial G1 arrest of MCF7 or U2OS cells in response to treatment with the PLK1 inhibitors, GSK461364 or BI6727, is dependent upon p53. Expression of p53 was silenced (or mock-silenced) in MCF7 cells (A, B) and U2OS cells (C, D), after which the cells were treated for 24 h with fixed concentrations of the drugs GSK461364 or BI6727 (or DMSO as control). The cells were subsequently harvested and analysed by flow cytometry (A, C) or western blotting (B, D). In panels A and C the arrows indicate the persistence of a subset of cells in G1 following drug treatment. The data are representative of two independent experiments, each done in triplicate. Error bars represent the standard deviation of the mean.

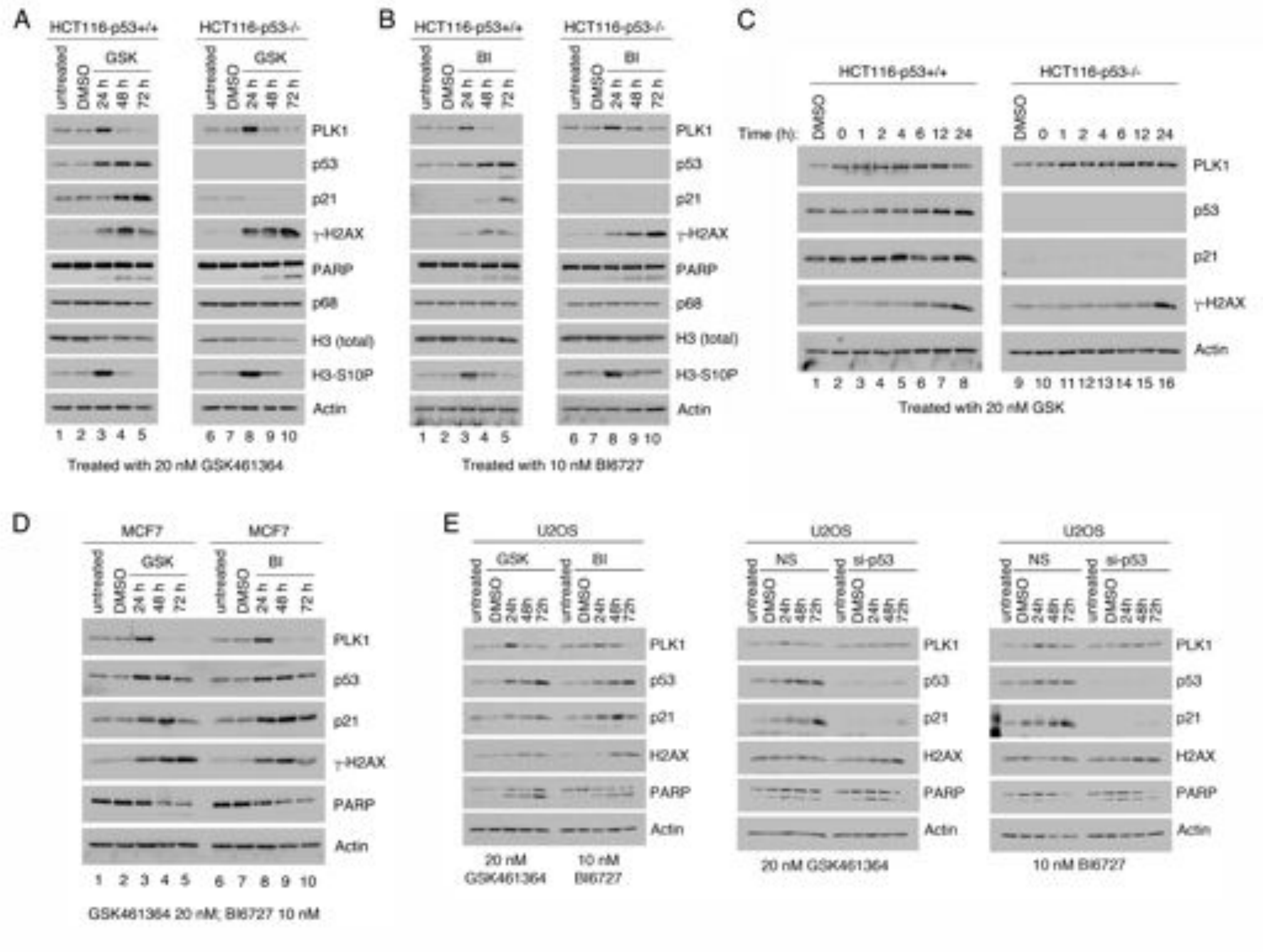

Supplementary Figure 2. The p53 pathway is induced following treatment with PLK1 inhibitors.
Various cell lines were treated with the PLK1-targeted drugs, GSK461364 or BI6727, for up to 72 h, followed by lysis and analysis by western blotting using the antibodies indicated in the figure. The cell lines were as follows: HCT116-p53+/+ and -p53-/- cells (A-C); MCF7 cells and U2OS cells (D,E respectively) previously treated with non-silencing or p53-targeted siRNAs.

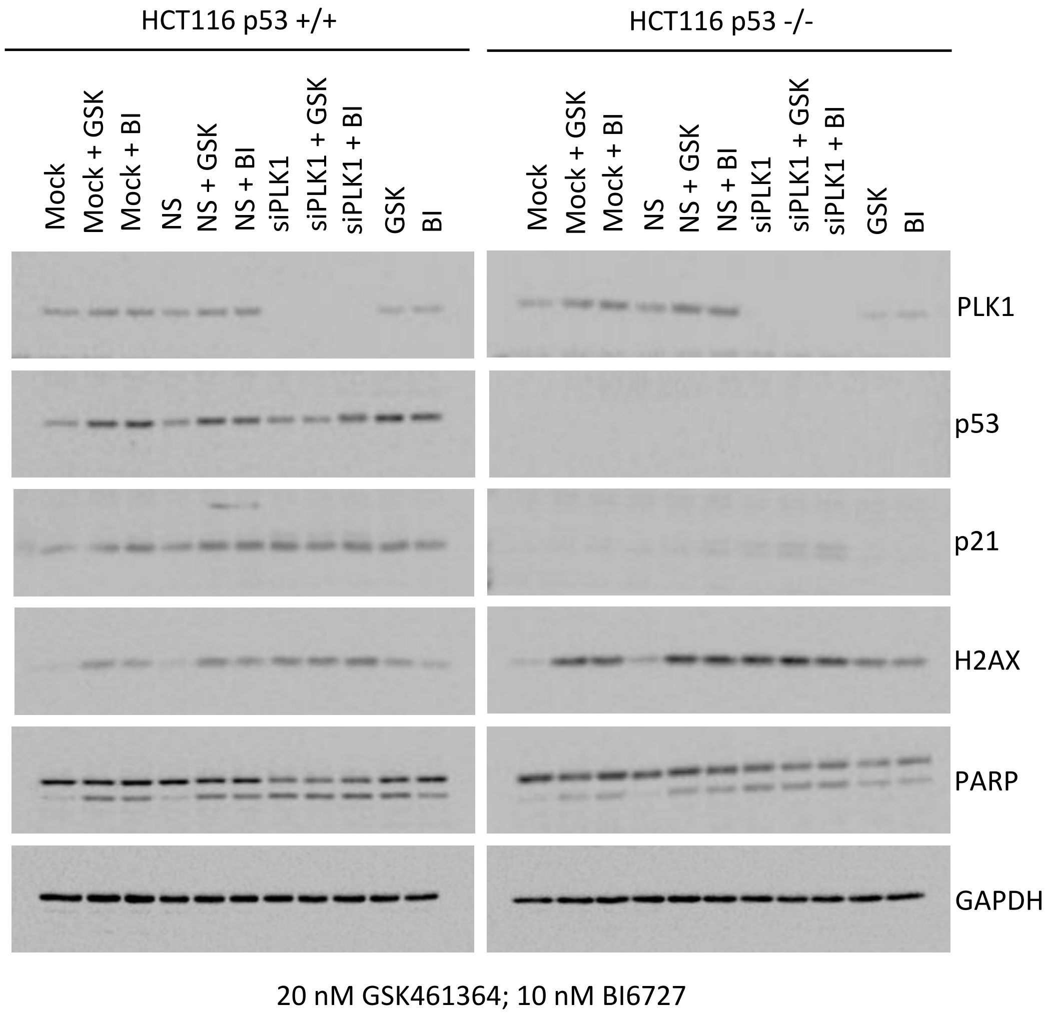

Supplementary Figure 3. Inhibition of PLK1 leads to DNA damage.
HCT116 p53+/+ and p53-/- cells were transfected with a mock transfection (transfection reagent alone), non-silencing siRNA or siRNA targeting PLK1. After 24 hours cells were treated with DMSO, 20 nM GSK461364 or 10 nM BI6727 for a further 24 hours. Cells were then harvested and western blotting was used with the antibodies indicated in the figure.

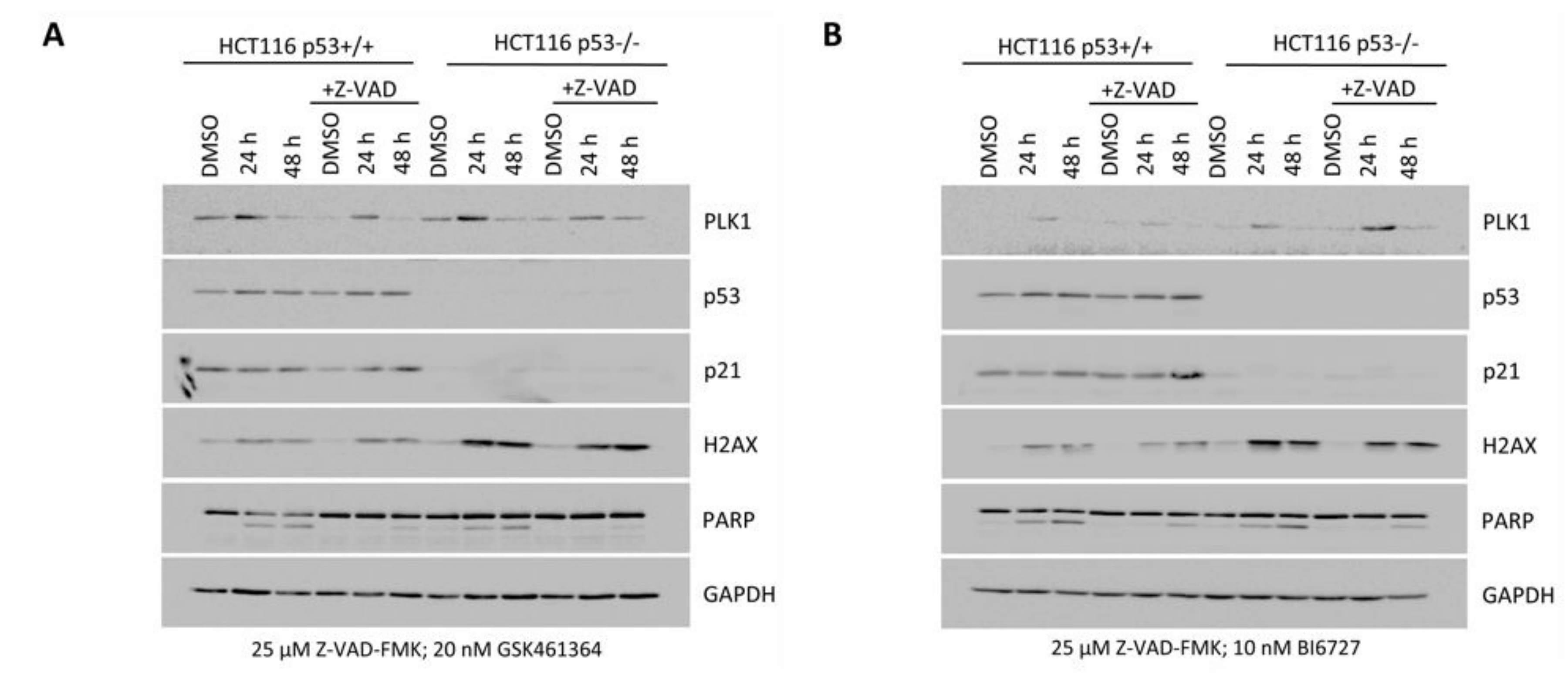

Supplementary Fig 4. The DNA damage is not caspase dependent.
HCT116 p53+/+ and p53-/- cells were pretreated for one hour with 25 µM Z-VAD-FMK or DMSO (vehicle control) before addition of (A) 20 nM GSK461364 or (B) 10 nM BI6727 for 24 or 48 hours. Cells were then harvested at the indicated time points and western blotting was used to assess protein levels using the antibodies shown in the figure.

A **MCF-7 p53 WT: treatment with GSK461364**

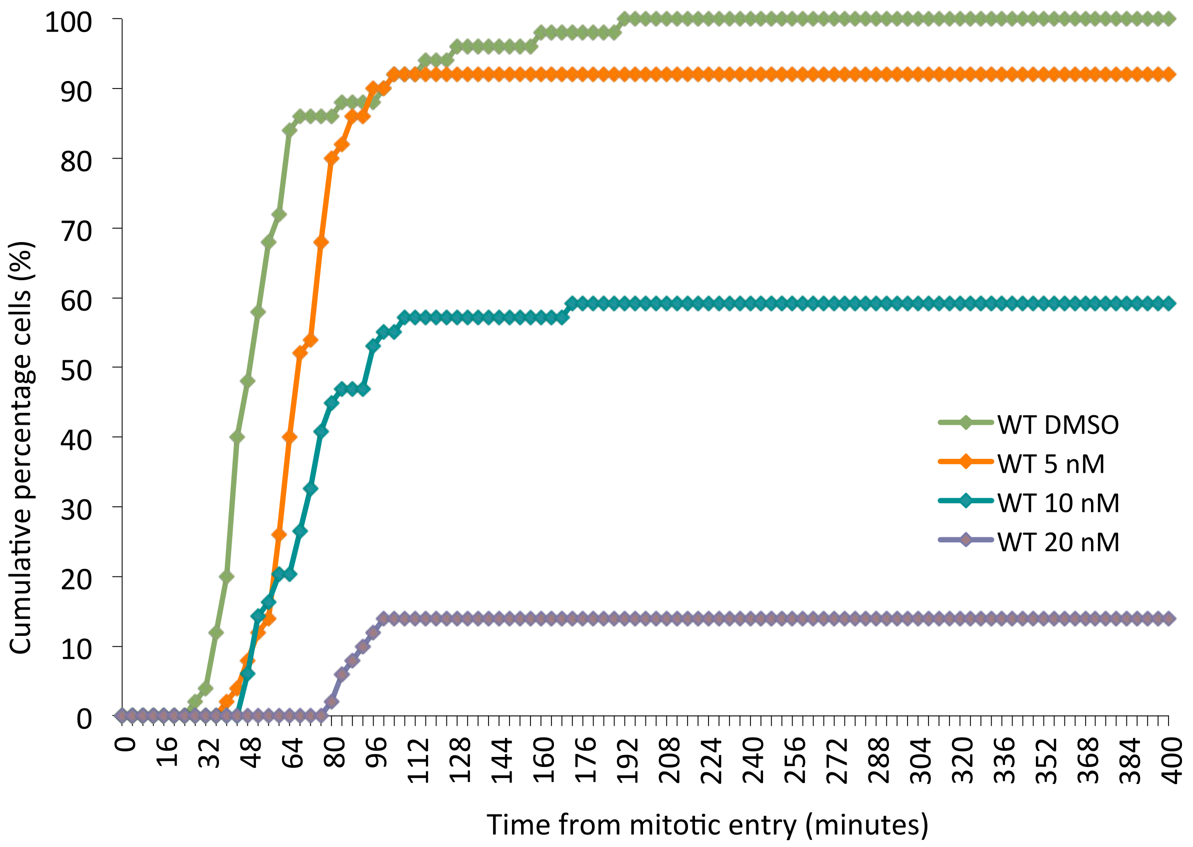

B **MCF-7 p53 KO: treatment with GSK461364**

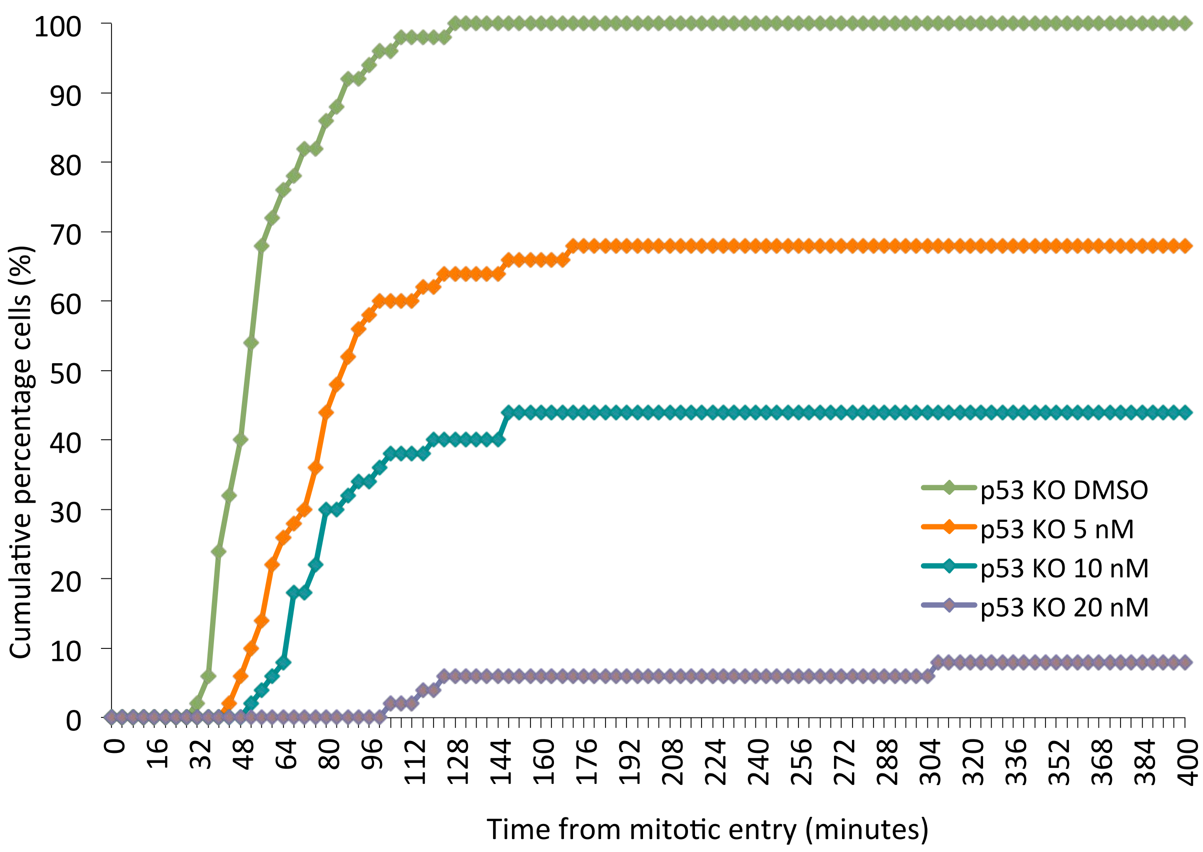

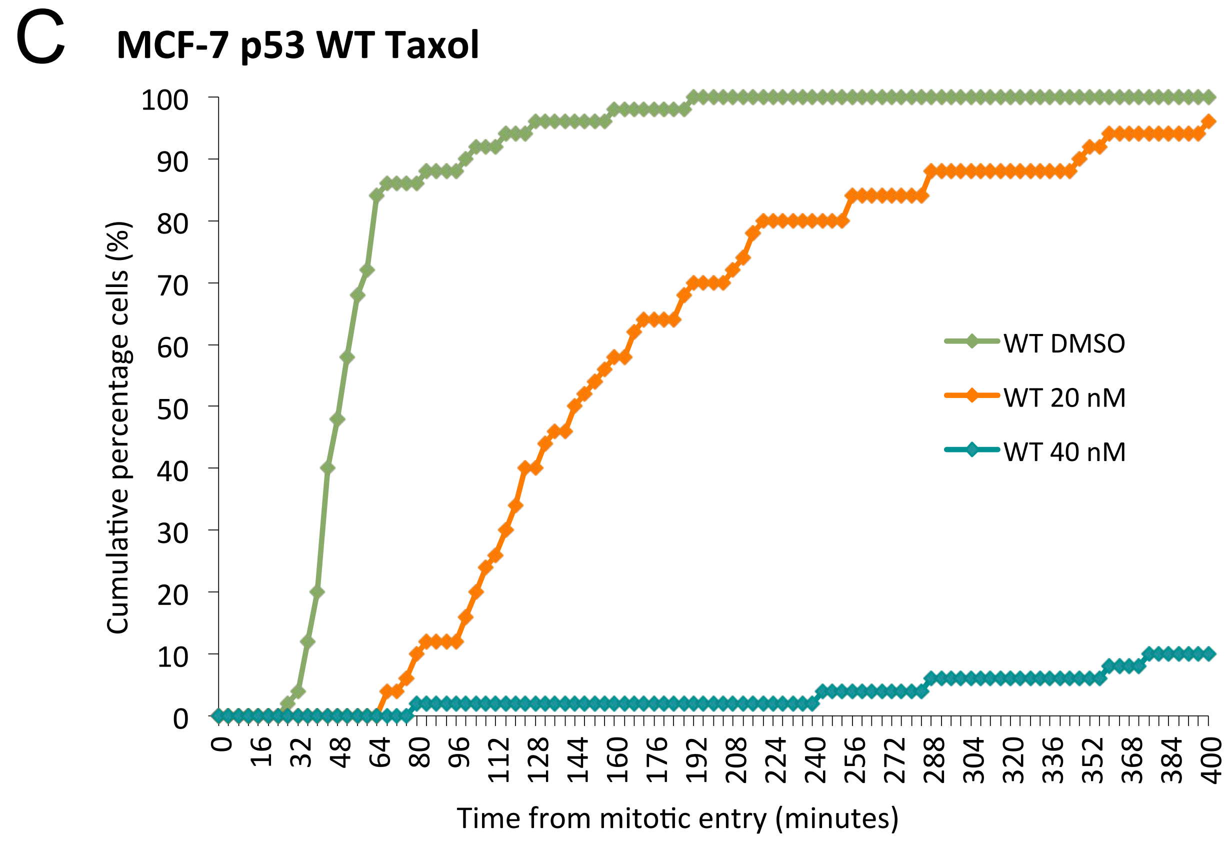

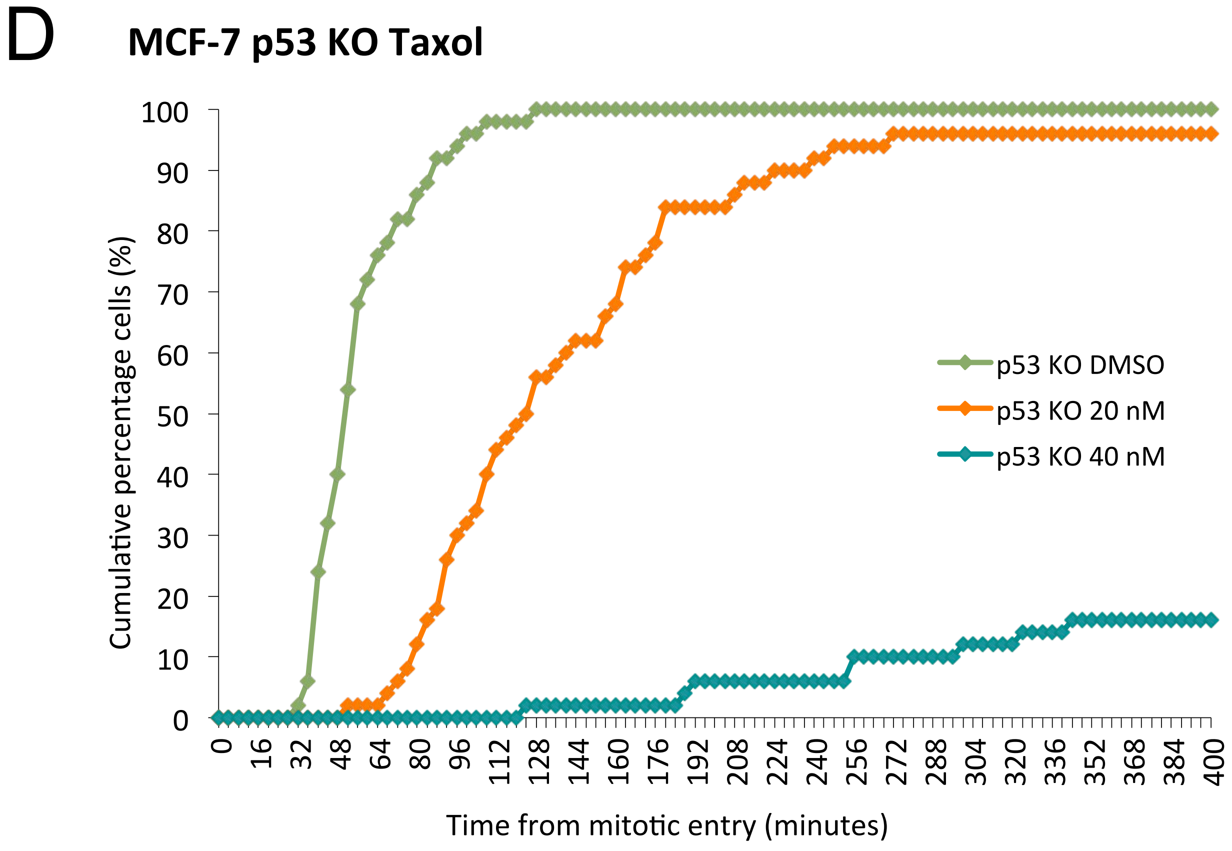

Supplementary Figure 5. p53 reduces the delay in mitosis of MCF7 cells resulting from inhibition of PLK1. MCF7 cells (A, C) and a derivative line in which p53 had been deleted using CRISPR (B, D) were treated with increasing concentraiton of GSK461364 (A, B) or taxol (C, D). Time-lapse microscopy analysis was then used to determine the duration of mitosis. The graphs represent the cumulative data from 50 cells for one experiment and are representative of three replicates.

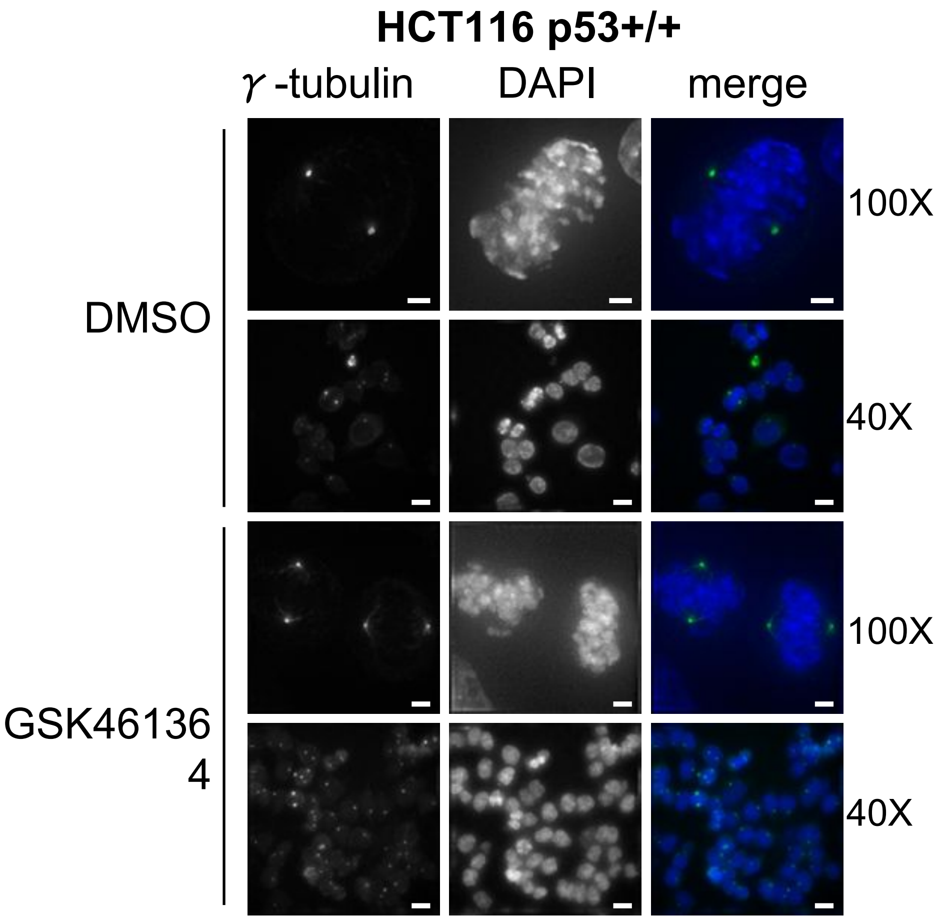
HCT116 p53+/+
γ-tubulin
DAPI
merge
DMSO
GSK46136
4
100X
40X
100X
40X

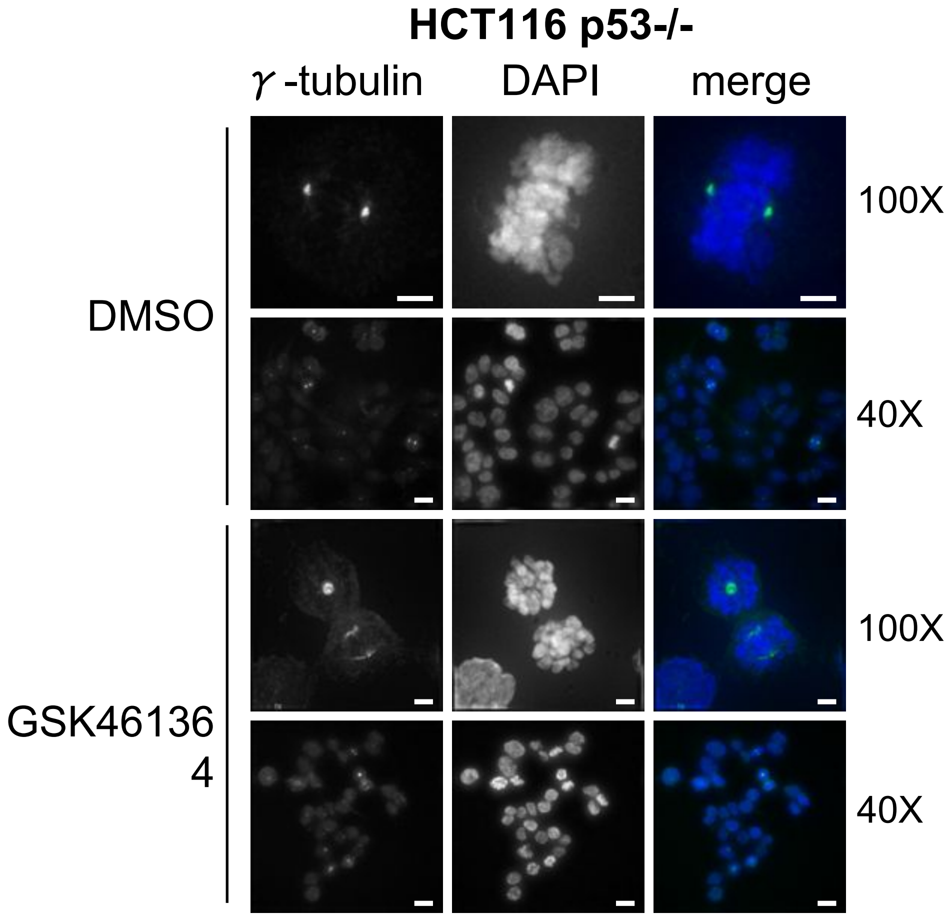
HCT116 p53-/-
γ-tubulin
DAPI
merge
DMSO
GSK46136
4
100X
40X
100X
40X

Supplementary Figure 6. p53 protects cells from inhibition of PLK1 by maintaining centrosome separation. HCT116 p53+/+ and p53-/- cells were treated with 20 nM GSK461364 or DMSO for 8 hours. Cells were subsequently fixed and stained with γ-tubulin antibody (a centrosome marker), followed by secondary staining with Alexa Fluor® 488 and DAPI. Fluorescence microscopy was then used to visualize mitotic cells showing a normal bipolar spindle (separated centrosomes) or monopolar spindle (non-separated centrosomes). Scale bars on the 40X magnification represent 10 microns, and on the 100X magnification represent 2 microns.

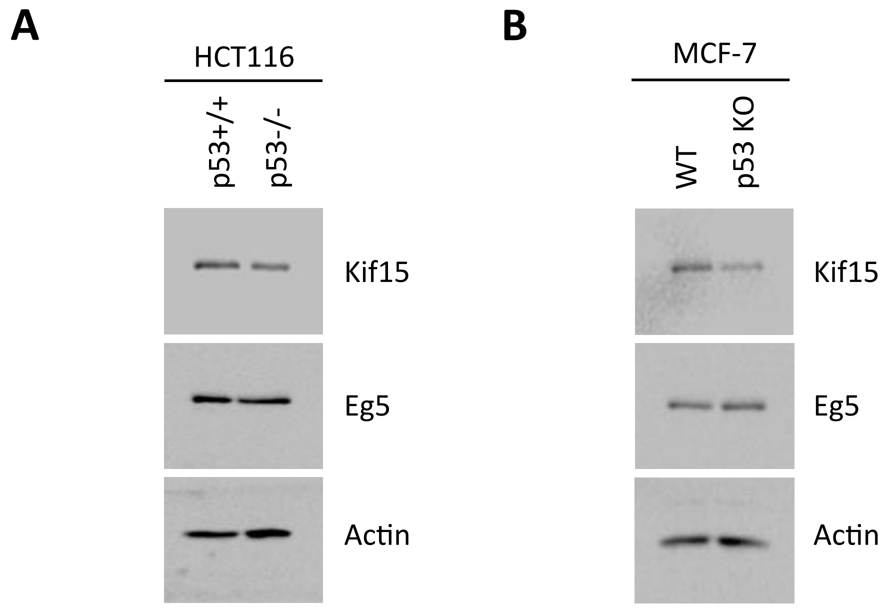

Supplementary Figure 7. Levels of Kif15 and Eg5 in p53competent and p53-knockout cells. Untreated HCT116 p53+/+ and p53-/- (A) and MCF-7 wild type and p53 knockout cells (B) were harvested and the cell lysate was analysed by western blotting.

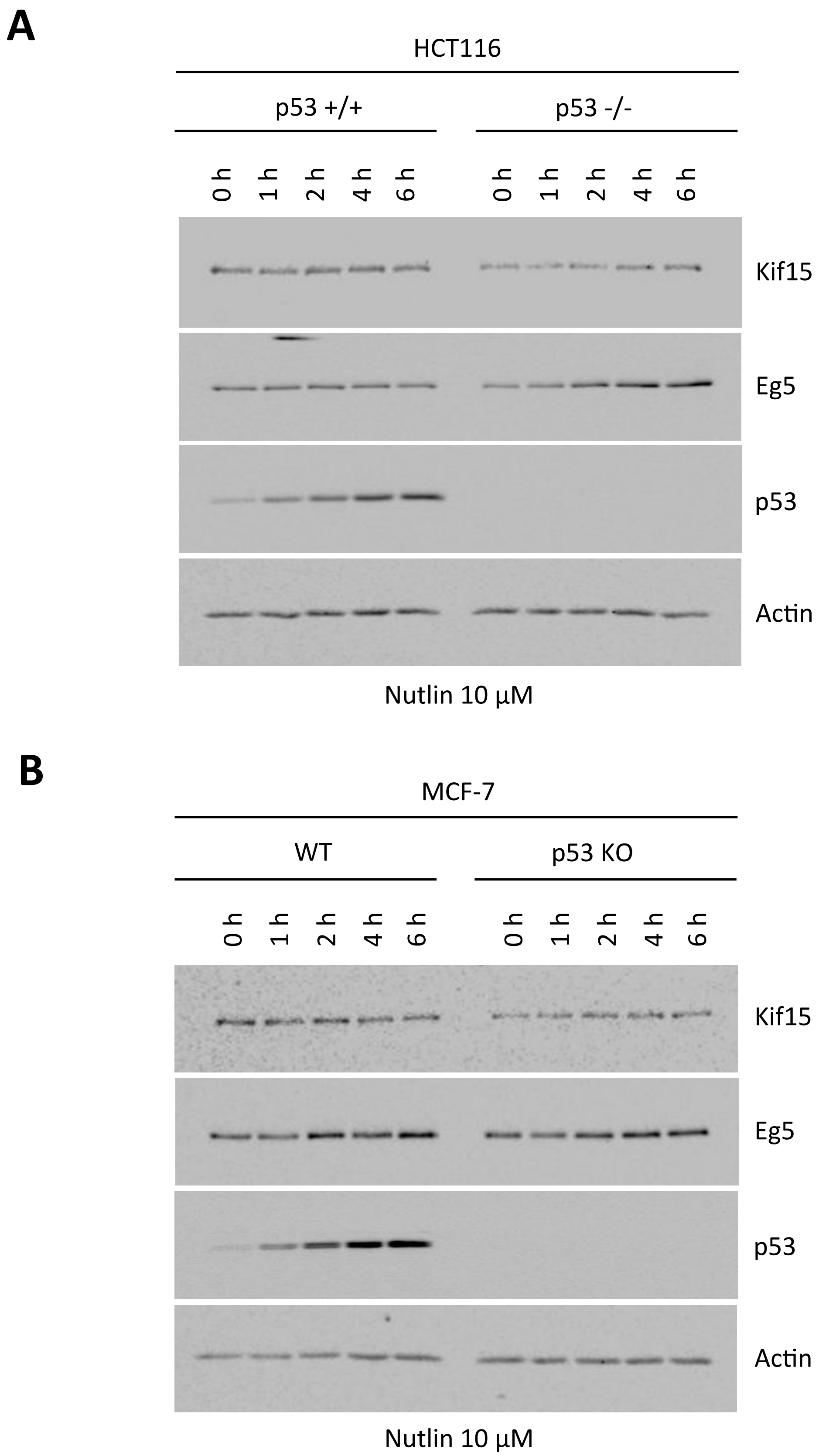

Supplementary Figure 8. Kif15 and Eg5 levels do not change upon treatment with Nutlin-3. HCT116 p53+/+ and p53-/- cells (A) or MCF-7 wild type and p53 knockout cells (B) were treated with 10 µM Nutlin-3 for 0, 1, 2, 4 or 6 hours. Cells were subsequently harvested, and analysed by western blotting with the antibodies indicated in the figure.

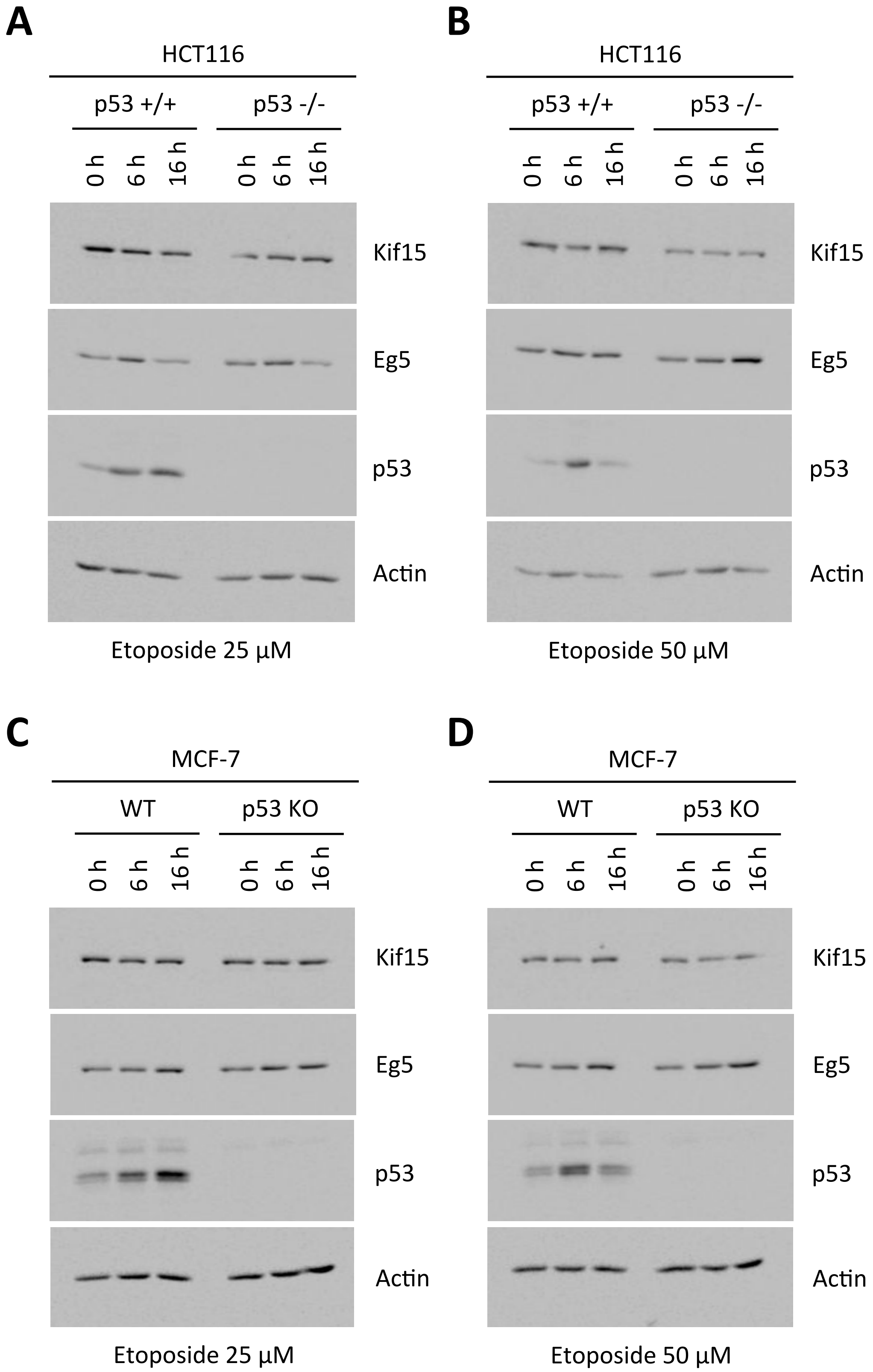

Supplementary Figure 9. Kif15 and Eg5 levels do not change upon treatment with etoposide. HCT116 p53+/+ and p53-/- (A+B) and MCF-7 wild type and p53 knockout cells (C+D) were treated with 25 µM (A+C) or 50 µM (B+D) etoposide. Cells were then harvested and analysed by western blotting with the antibodies indicated in the figure.

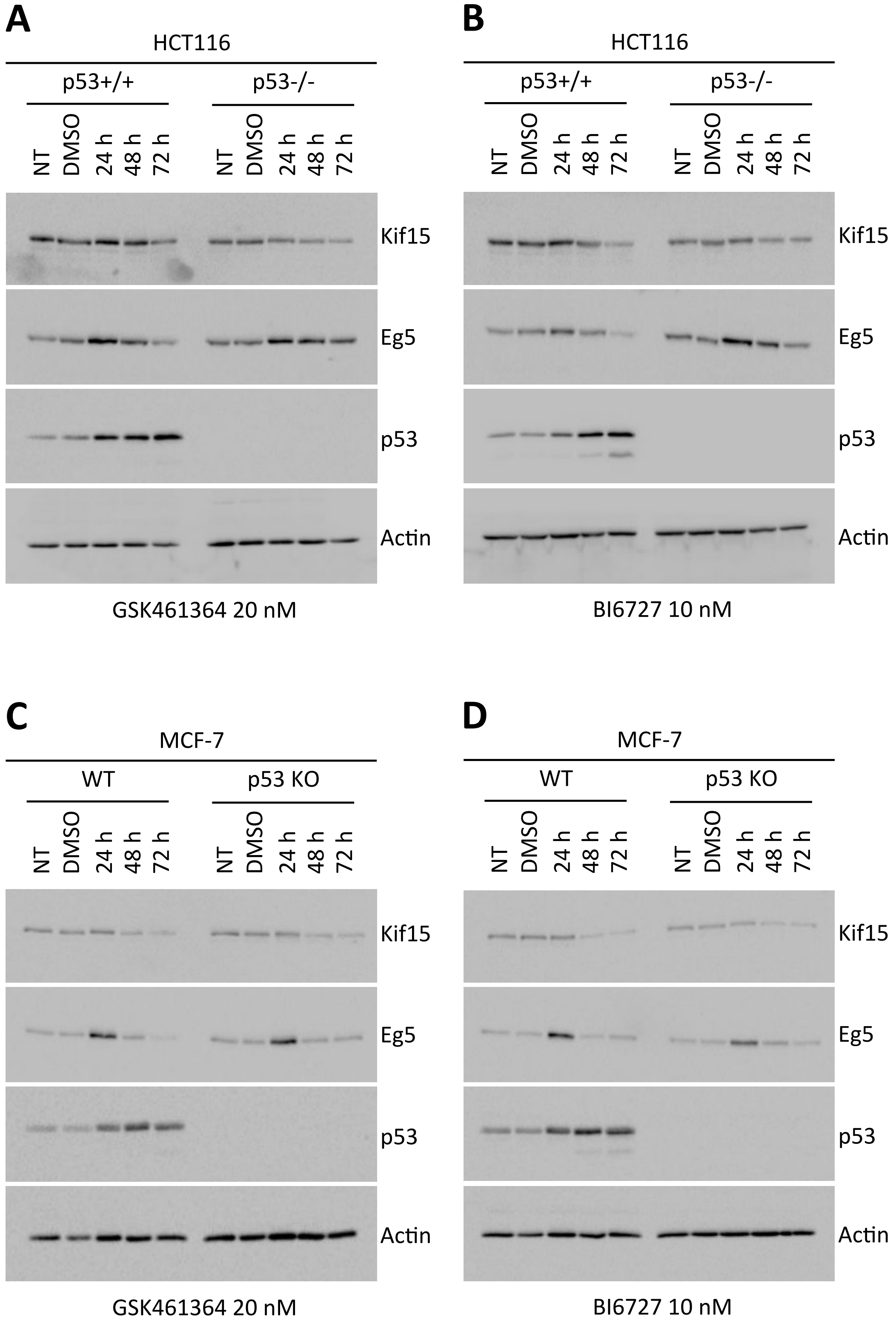
A
HCT116
p53+/+
p53-/-
NT
DMSO
24 h
48 h
72 h
Kif15
Eg5
p53
Actin
GSK461364 20 nM
B
HCT116
p53+/+
p53-/-
NT
DMSO
24 h
48 h
72 h
Kif15
Eg5
p53
Actin
BI6727 10 nM
C
MCF-7
WT
p53 KO
NT
DMSO
24 h
48 h
72 h
Kif15
Eg5
p53
Actin
GSK461364 20 nM
D
MCF-7
WT
p53 KO
NT
DMSO
24 h
48 h
72 h
Kif15
Eg5
p53
Actin
BI6727 10 nM

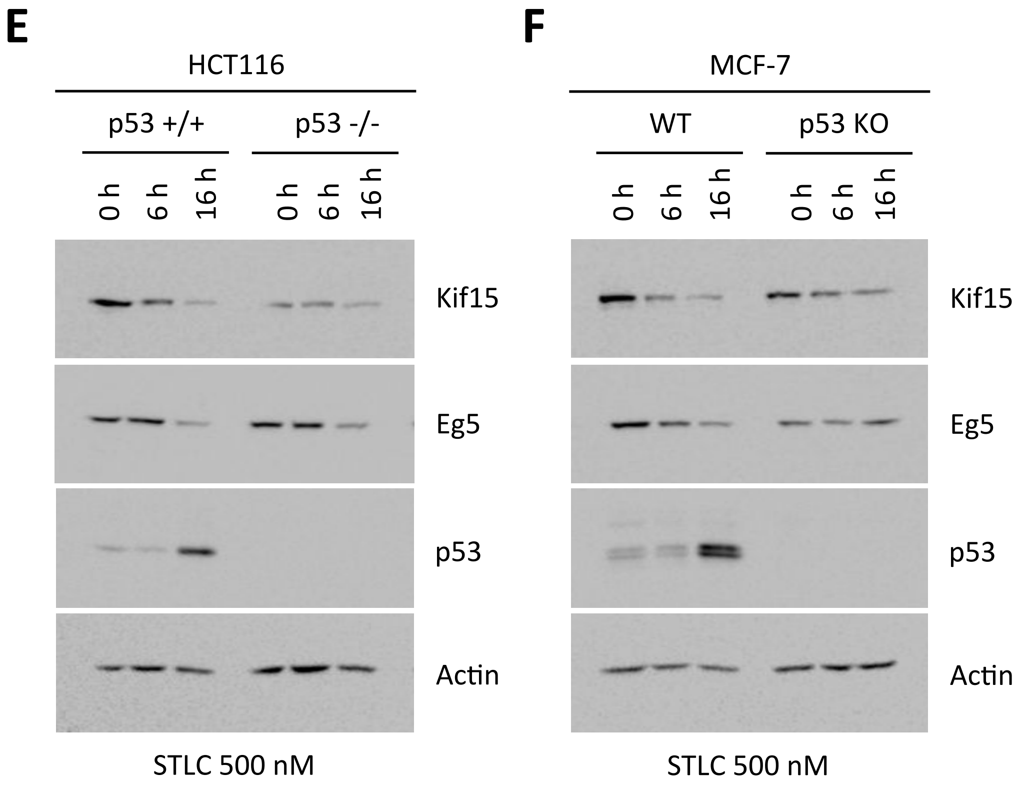

Supplementary Figure 10. Kif15 and Eg5 levels upon treatment with PLK1 and Eg5 inhibitors. HCT116 p53+/+ and p53-/- cells (A,B+E) and parental MCF-7 and p53 knockout cells (C,D+F) were treated with PLK1 inhibitors, GSK461364 (A+B) and BI6727 (C+D), and Eg5 inhibitor, STLC (E+F). PLK1 inhibitors were applied for 24, 48 or 72 hours, along with an untreated and DMSO treated sample, whilst STLC was applied for 0, 6 or 16 hours. After harvesting, cell lysates were analysed by western blotting using the antibodies indicated in the figure.

Supplementary Movies 1-4. Cells lacking p53 are delayed in mitosis following treatment with GSK461364. HCT116-p53+/+ and -p53-/- cells were stained with SiR-DNA (SiR-Hoechst*) and treated with DMSO or 20 nM GSK461364. Cells were mounted on the Deltavision Elite live cell microscope and imaged using the Cy5 (far red) channel and in phase contrast every 4 minutes over 250 time points. The movies are as follows:

Movie 1: HCT116-p53+/+ untreated
Movie 2: HCT116-p53-/- untreated
Movie 3: HCT116-p53+/+ plus GSK461364
Movie 4: HCT116-p53-/- plus GSK461364

Supplementary Movies 1-4. Cells lacking p53 are delayed in mitosis following treatment with GSK461364. HCT116-p53+/+ and -p53-/- cells were stained with SiR-DNA (SiR-Hoechst*) and treated with DMSO or 20 nM GSK461364. Cells were mounted on the Deltavision Elite live cell microscope and imaged using the Cy5 (far red) channel and in phase contrast every 4 minutes over 250 time points. The movies are as follows:

Movie 1: HCT116-p53+/+ untreated
Movie 2: HCT116-p53-/- untreated
Movie 3: HCT116-p53+/+ plus GSK461364
Movie 4: HCT116-p53-/- plus GSK461364

FULL LENGTH GELS FOR FIG 4A

Gels were cut to sizes below prior to probing

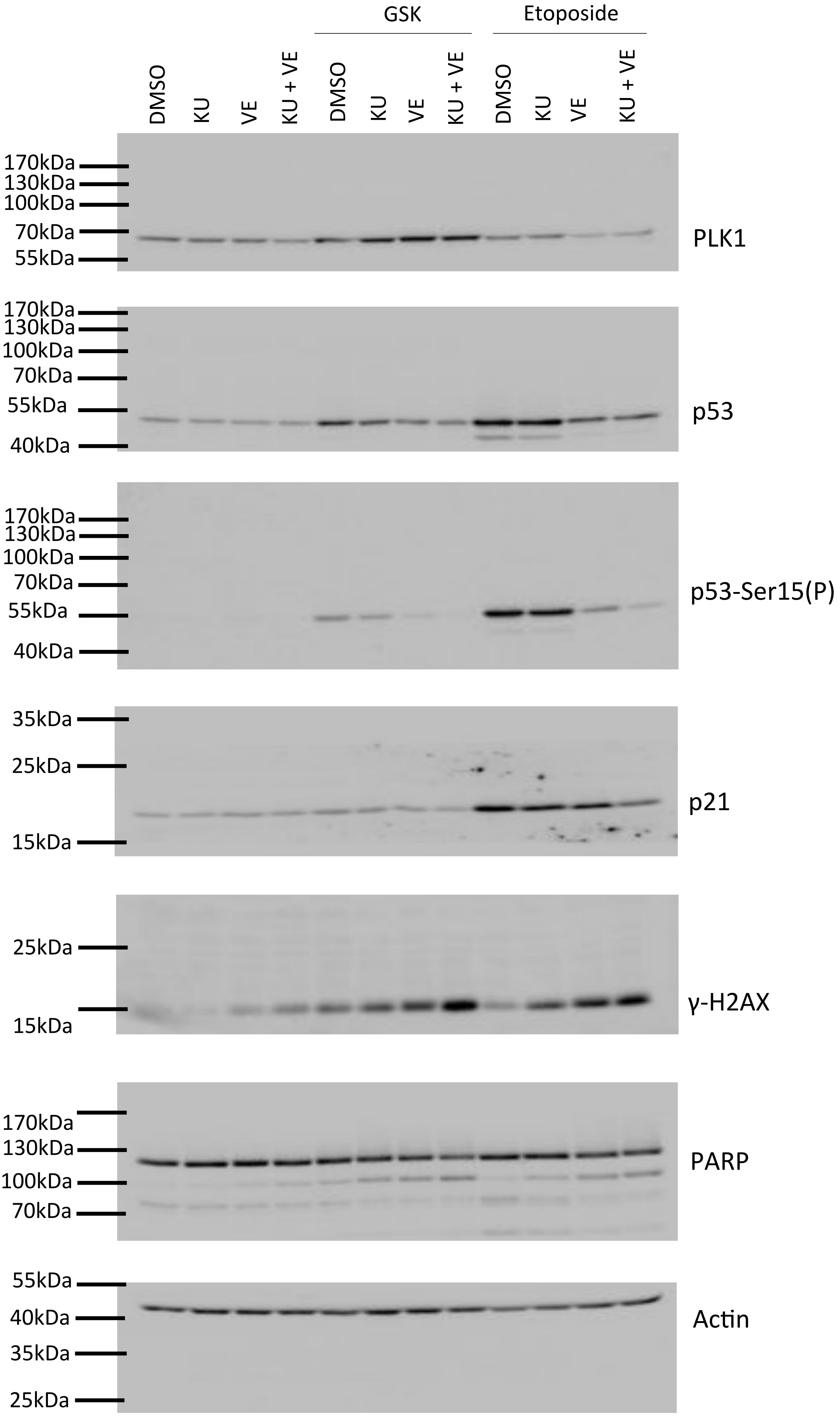

FULL LENGTH GELS FOR FIG 4C

p53/p21 gel was cut prior to probing

p53

p21

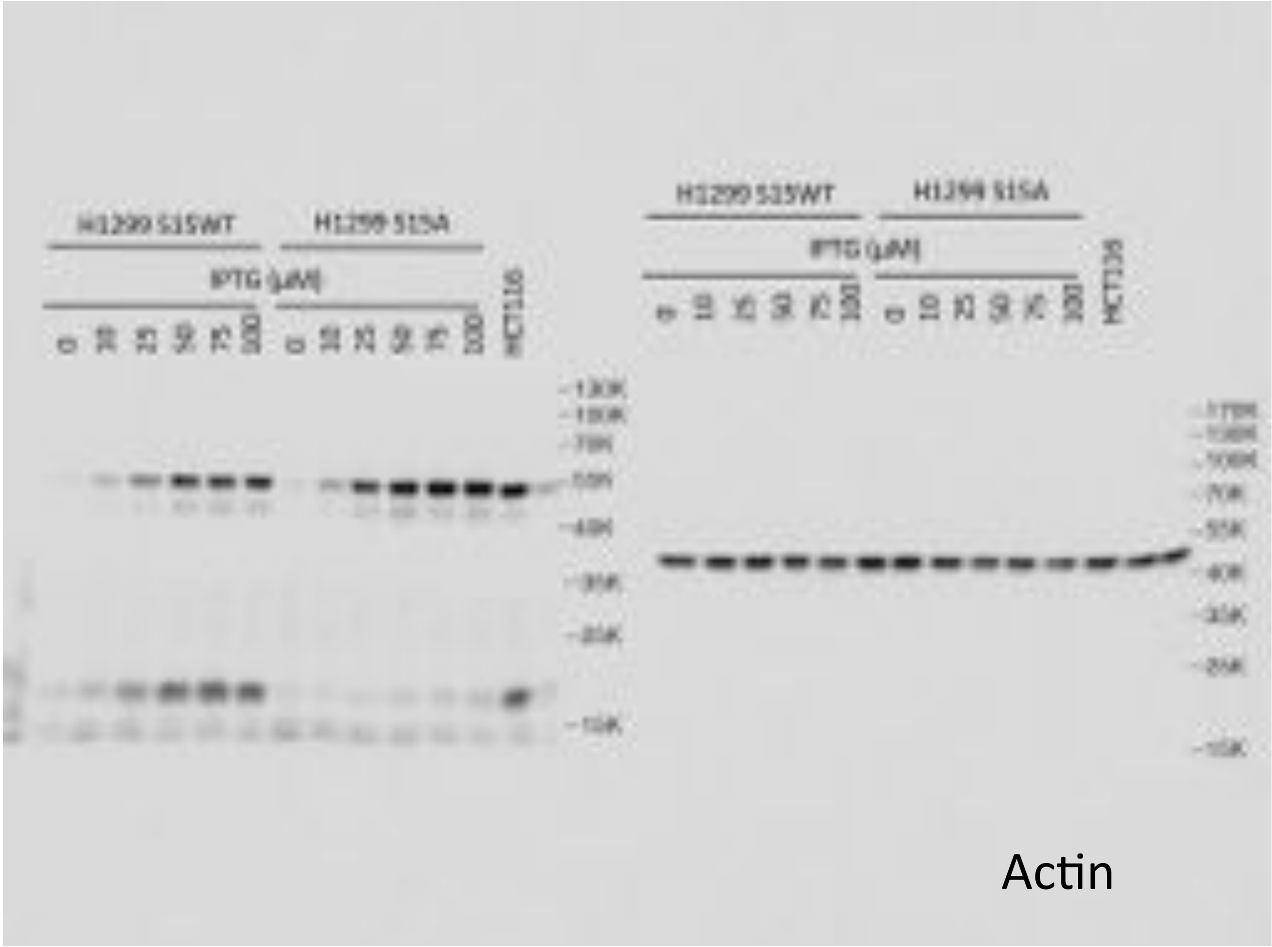

Supplement: Supplementary file 1 — Supplementary Information [file 41598_2017_16394_MOESM1_ESM.pdf]
